# Towards a quantitative theory for transmission X-ray microscopy

**DOI:** 10.3762/bjnano.16.82

**Published:** 2025-07-15

**Authors:** James G McNally, Christoph Pratsch, Stephan Werner, Stefan Rehbein, Andrew Gibbs, Jihao Wang, Thomas Lunkenbein, Peter Guttmann, Gerd Schneider

**Affiliations:** 1 Helmholtz-Zentrum Berlin für Materialien und Energie, Elektronenspeicherring BESSY II, Albert-Einstein-Straße 15, 12489 Berlin, Germany; 2 Department of Mathematics, University College London, London, UK; 3 Department of Inorganic Chemistry, Fritz-Haber-Institut der Max-Planck Gesellschaft, Berlin, Germany; 4 Humboldt Universität zu Berlin, Mathematisch-Naturwissenschaftliche Fakultät, Institut für Physik, Newtonstraße 15, 12489 Berlin, Germany

**Keywords:** 3D imaging, mathematical model, Mie theory, nanoparticle, transmission X-ray microscope

## Abstract

Transmission X-ray microscopes (TXMs) are now increasingly used for quantitative analysis of samples, most notably in the spectral analysis of materials. Validating such measurements requires quantitatively accurate models for these microscopes, but current TXM models have only been tested qualitatively. Here we develop an experimental and theoretical framework for evaluation of TXMs that uses Mie theory to compute the electric field emerging from a nanosphere. We approximate the microscope’s condenser illumination by plane waves at the mean illumination angle and the zone plate by a thin lens. We find that this model produces good qualitative agreement with our 3D measurements of 60 nm gold nanospheres, but only if both β and δ for the complex refractive index *n* = 1 – δ + *i*β of gold are included in the model. This shows that both absorption and phase properties of the specimen influence the acquired TXM image. The qualitative agreement improves if we incorporate a small tilt into the condenser illumination relative to the optical axis, implying a small misalignment in the microscope. Finally, in quantitative comparisons, we show that the model predicts the nanosphere’s expected absorption as determined by Beer’s law, whereas the microscope underestimates this absorption by 10–20%. This surprising observation highlights the need for future work to identify the microscope feature(s) that lead to this quantitative discrepancy.

## Introduction

Transmission X-ray microscopes (TXMs) operating in the soft and tender X-ray energy range are valuable tools for structural analysis in both biomedical and materials science research [1–4]. These microscopes yield images at a lateral resolution approaching 25 nm, from which quantitative data are often extracted. For example, in materials science applications, nanoscale spectromicroscopy [5–8] is used to examine a sample around its absorption edges, which provides insights into its electronic structure. In biomedical applications [9–12], absorption at selected locations in the sample is used to determine the amount of a material at those sites.

Validation of these quantitative measurements requires a quantitatively accurate TXM model. Such models are also valuable both for the design of new soft TXMs, which relies on a deep understanding of the microscope’s image-formation process, and for the improvement of 3D tomographic reconstruction procedures [13–16], which are used to produce 3D volumes from a 2D tilt series of TXM images. Over the past three decades, models for soft TXMs have grown increasingly sophisticated [17–19], with the two most recent both incorporating the 3D nature of the imaging process [20–21]. A 3D model is essential to accurately account for the microscope’s large but nevertheless finite depth of focus. Otón et al. [20] developed the first 3D model, making several assumptions to simplify the analysis, including that imaging is incoherent and that only the absorption component β of the sample’s refractive index needs to be considered. The latter assumption means that light rays traversing the sample follow Beer’s law of absorption, and so we refer to this model as the incoherent Beer’s law (inc-BL) model.

Not all the assumptions of the inc-BL model match the TXM we have used in this study at the electron storage ring BESSY II in the Helmholtz-Zentrum Berlin. Specifically, the sample illumination is not incoherent, but rather partially coherent because the sample is illuminated over a narrow angular range of 0.83–1.18° and the numerical aperture (NA) of the condenser is less than the NA of the zone plate objective.

To improve the inc-BL model, Selin et al. [21] developed a more sophisticated 3D model that addressed the microscope’s annular illumination by replacing the full annular illumination range by its mean angle. This mean angle is less than the acceptance angle of the objective and so introducing this into the model also accounts for the microscope’s partial coherence arising from the mismatch of the condenser and objective NA’s. Furthermore, rather than Beer’s law, Selin et al. used the parabolic wave equation to propagate light through a sample that was defined by its 3D distribution of complex refractive indices, thereby accounting for both the absorption and phase properties of the sample. We refer to this model as the partially coherent, parabolic-wave-equation (pc-PWE) model.

In the current study, we continue this effort toward further development of 3D models for TXMs. First, we introduce Mie theory as an alternate approach for light propagation through the sample. Mie theory provides an exact solution to Maxwell’s equations but limited to spheres of known refractive index [22] and so is well suited to the gold nanospheres that we use as test objects in this study. We therefore name our model the partially coherent Mie (pc-Mie) model. Second, we compare the theory for our model to the preceding two 3D models, providing a deeper understanding of the principles that underlie the different models. Third, we perform detailed qualitative comparisons of the different models’ predictions to the measured data from the gold nanosphere. This qualitative analysis demonstrates the importance of accounting for both β and δ in the sample’s refractive index, and it also identifies a tilt in the optical train that requires adjustment of the model. Finally, we perform the first quantitative comparisons of a 3D model to real data, revealing that the microscope underestimates the absorption of the 60 nm gold nanospheres.

In sum, our work not only extends the modelling framework for TXMs but also identifies two areas for future work that will improve the quantitative analysis of TXM data. First, our finding that both β and δ play a role in TXM imaging highlights the need to investigate to what extent δ influences quantitative analyses such as those in spectromicroscopy that currently presume a role only for β. Second, our surprising finding that the microscope underestimates absorption underscores the need to understand how this discrepancy arises such that more accurate quantitative TXM measurements are possible.

## Results

### 1 Overview: microscope configuration

To understand the various 3D microscope models and their assumptions, we first describe the key features of the BESSY II soft TXM. The microscope’s X-ray source begins at the undulator, which produces largely incoherent light (<2% coherence for the BESSY II third generation synchrotron, p. 32 in [23]). This X-ray light is then propagated through a beamline about 26 m in length [24], which consists of several mirrors and a plane-grating monochromator that yields highly monochromatic light (*E*/Δ*E* ≈ 10000 for the energy of 510 eV used in the current study).

**Figure 1:**
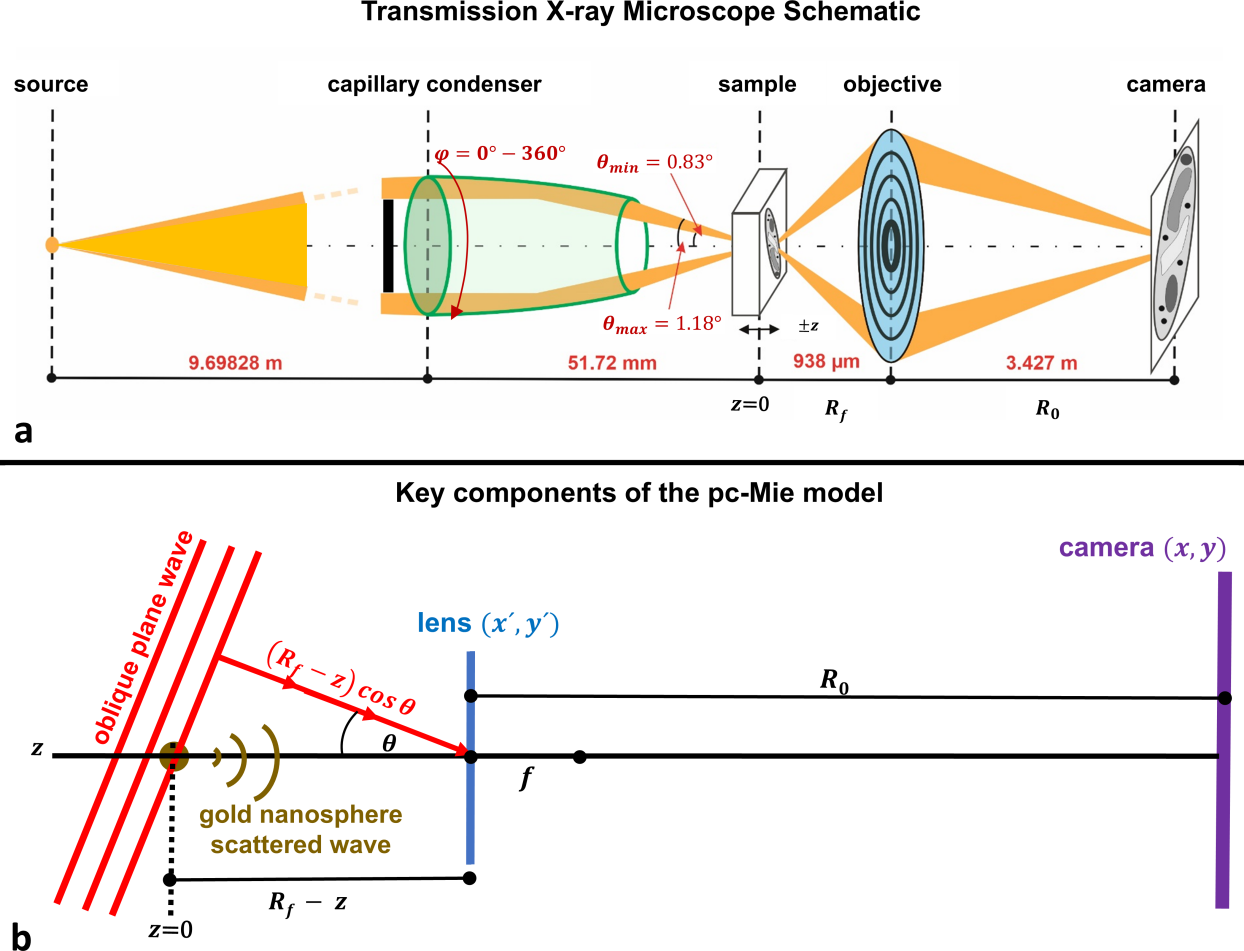
(a) Schematic of the Helmholtz-Zentrum Berlin TXM at the electron storage ring BESSY II. X-ray light from the focal spot produced by the monochromator beamline is demagnified and re-focused by the capillary condenser (which is an ellipsoidal mirror) onto the sample plane and then imaged by a zone-plate objective onto the camera plane. Distances between the different planes for operation at 510 eV are indicated in red. A central stop (black bar) is present in front of the ellipsoidal mirror to prevent zero-order light from reaching the zone plate. The minimum and maximum polar angles (θ) of the condenser illumination are also indicated in red, as is the full circumferential range of azimuthal angle (φ) illumination. (b) The pc-Mie model with key variables shown is based on an oblique plane wave emanating from the condenser that induces a scattered wave in the nanosphere, whose field is calculated using Mie theory. Both the plane and scattered waves are then propagated through the zone plate, which is treated as a lens, and then to the camera (not to scale).

This monochromator source is then re-focused and demagnified onto the sample plane by a truncated ellipsoidal glass capillary that acts as an ellipsoidal mirror [4,25] positioned at ≈9.7 m from the monochromator exit slit. A central stop is present in front of this ellipsoidal mirror to prevent zero-order light from passing directly through the objective. This combination of the ellipsoidal mirror and central stop produces illumination of the sample (Figure 1a) over an angular range of 0.83–1.18°.

The final size of the ellipsoidal mirror’s focused spot (≈2.7 μm × 0.7 μm) is smaller than most samples, and therefore to generate an image of the sample, the focused spot from the ellipsoidal mirror is “scanned” by moving the mirror in a spiral pattern. This light then reaches the microscope’s objective, which is a zone plate composed of 900 zones with outermost zone width of *dr*_n_ = 25 nm. Finally, images from the objective are acquired with a CCD camera.

### 2 Models: partially coherent Mie, partially coherent parabolic wave equation, and incoherent Beer’s law

We introduce our new model (pc-Mie) and compare it to the other two 3D TXM models (pc-PWE [21] and inc-BL [20]). As in [21], our description of each model is divided into three steps: (1) illumination of the sample by the condenser; (2) light propagation through the sample to the zone plate; and (3) light propagation from the zone plate to the camera. The key features of each model and their assumptions are summarized in Table 1.

**Table 1:** Comparison of the existent 3D soft TXM models to the actual Helmholtz-Zentrum Berlin TXM.^a^

	BESSY II Microscope	Model 1: pc-Mie	Model 2: pc-PWE	Model 3: inc-BL

illumination	annular angles (0.83–1.18°)	mean annular angle (1.01°)^b^	mean annular angle (1.01°)^b^	all angles (0–90°)^b^
coherence	partial	partial	partial	incoherent^b^
coherence patch size	unknown	small^b^	small^b^	na^b^
spiral scan pattern	yes	no^b^	no^b^	no^b^
sample *n*	absorption (β) phase (δ)	absorption (β) phase (δ)	absorption (β) phase (δ)	absorption (β) no phase (δ = 0)^b^
light propagation	Maxwell’s equations	Mie theory	parabolic wave equation^b^	Beer’s law^b^
objective	zone plate	lens^b^	lens^b^	lens^b^

^a^pc = partially coherent; PWE = parabolic wave equation; inc-BL = incoherent Beer’s law; na = not applicable; ^b^features that are approximations to the actual microscope.

#### 2.1 Illumination of the sample by the condenser

Neither of the preceding 3D models considered the spiral scanning of the condenser spot over the sample. This is a reasonable first approximation since the scanning process can be viewed as producing a patchwork of images that are then added to produce the final acquired image, which should not be significantly different from the image that would be produced from a much larger condenser spot, and so we have also not incorporated the scanning into our model.

The different models make different assumptions about the polar angles at which the sample is illuminated, and this relates to whether the model is incoherent or partially coherent. By assuming incoherent illumination, the inc-BL model implicitly incorporates illumination over all possible azimuthal angles (φ = 0–360°) spanning the ellipsoidal mirror circumference and over all possible polar angles (θ = 0–90°). This model therefore ignores the limited annular polar angle range of the true condenser (0.83–1.18°). In the pc-PWE model, the sample is also illuminated from the complete range of azimuthal angles φ = 0–360°, but at a constant polar angle θ_m_ that corresponds to the mean angle of the condenser’s annular illumination range (θ_m_ = 1.01° in our case, Supporting Information File 1, Section S1). We adopt the same assumption in our pc-Mie model of a mean polar illumination angle.

Since the different polar angles of illumination are related to the assumptions of either incoherence or partial coherence, these in turn affect whether fields or intensities are added to produce the final image. The inc-BL model assumes incoherence and therefore calculates the intensities arising at each point in the sample. Both the pc-PWE and pc-Mie model are partially coherent because they include the mean polar angle of annular illumination and so intrinsically account for the mismatch of condenser and objective NA. These partially coherent models must therefore also specify the size of a coherence domain over which field amplitudes are added before computing intensities.

The coherence patch size arising from our beamline illumination has not been measured in our TXM. The pc-PWE model assumed that this size was small, and as discussed later, we also find that to match our acquired data, the coherence patch size must be small. Future measurements of the actual coherence patch size of our TXM will be important to determine whether the measured size matches the predictions of a small patch predicted by both our model and the pc-PWE model.

Finally, all three models also assume that the sample illumination can be approximated as plane waves impinging on the sample at the corresponding polar and azimuthal angles (Figure 1b). This is an excellent approximation as demonstrated by modeling the ellipsoidal mirror as an annular lens that produces a converging spherical wave at its focal point in the sample plane. An angular spectrum decomposition of this lens field shows that it is very accurately described as a sum of plane waves spanning the polar angular range (Supporting Information File 1, Section S2).

#### 2.2 Propagation of light through the sample to the zone plate

We first consider light propagation through the sample in our pc-Mie model and then proceed to a discussion of light propagation in the other two 3D models. Mie theory provides an exact solution to Maxwell’s equations for a sphere of given diameter and homogeneous refractive index *n* = 1 – δ + *i*β [22], an approach well suited to the spherical nanoparticles examined here.

Mie theory is one example of scattering theory in which the time-independent part of the final field is given by the sum of the incident and scattered fields:


[1] $$U\left(r\right)=U_{\text{i}}\left(r\right)+U_{\text{s}}\left(r\right).$$

At a sufficient distance from the scattering object (in our case, the distance between the nanosphere and the zone plate), the scattered field is well approximated by a spherical wave, and can be written as:


[2] $$U_{s}\left(r\right)=\frac{S\left(\psi \right)}{\left(-ik\right)}\left[\frac{\exp\left(ik\left|r\right|\right)}{\left|r\right|}\right],$$

where **S** is the scattering amplitude, ψ the scattering angle from the sphere to a point in the zone plate, and *k* the wave number. When the incident field **U**_i_(**r**) is a plane wave of amplitude one propagating along the optical axis (*z*_p_ where the subscript “p” indicates this is a propagation distance), then Equation 1 and Equation 2 yield the total field at the zone-plate plane:


[3] $$U\left(r\right)=\exp\left(ikz_{\text{p}}\right)+\frac{S\left(\psi \right)}{\left(-ik\right)}\left[\frac{\exp\left(ik\left|r\right|\right)}{\left|r\right|}\right],$$

and the problem reduces to determination of the scattering amplitude **S**(ψ).

The Mie solution **S**(ψ) is given by an infinite series of vector spherical harmonics that for numerical computations is truncated based on the accuracy required. Conventional Mie theory is based on plane waves parallel to the optical axis, and we adapt this by applying a simple geometrical transformation (Supporting Information File 1, Section S3) to account for oblique plane waves emerging from the ellipsoidal-mirror condenser. This yields an expression for the scattered field **S**_θ_(*x*′,*y*′) arising due to an oblique plane wave at a polar angle θ to the optical axis and propagating through a sphere to an arbitrary point (*x*′,*y*′) in the zone plate. To compute **S**, we have used Matlab code developed by Mätzler [26].

The scattering amplitude **S**(ψ) is a vector with two components, S_1_(ψ) and S_2_(ψ). For scattering at small angles, the Mie theory reduces to a simplified scalar theory known as the anomalous diffraction approximation (ADA) [27] with S_1_(ψ) = S_2_(ψ) = S_ADA_(ψ) given by:


[4] $$S_{\text{ADA}}\left(\psi \right)=k^{2}\int_{0}^{a}\left(1-e^{2ik\left(n-1\right)\sqrt{a^{2}-\xi^{2}}}\right)J_{0}\left(k\xi \psi \right)\xi \text{d}\xi ,$$

where ξ is the radial coordinate in the sphere and *J*_0_ the Bessel function of the first kind. This equation reflects the sum of the optical path length along different rays parallel to the *z*-axis passing through the nanosphere relative to rays passing outside the nanosphere [27].

This anomalous diffraction approximation holds under two conditions that should apply in our experiments, namely when the wavelength λ is small relative to the diameter 2*a* of the sphere, and when the magnitude of the complex refractive index *n* is close to one [27]. These two requirements ensure that light rays pass through the sphere without significant deflection or reflection, subject only to the accumulated phase change. We confirm the validity of the anomalous diffraction approximation for 60 nm gold nanospheres imaged with 2.4 nm light in Supporting Information File 1, Section S4, where we demonstrate that the full Mie solution for either S_1_(ψ) or S_2_(ψ) is identical to the anomalous diffraction solution S_ADA_(ψ).

We then show in Supporting Information File 1, Section S5, how the anomalous diffraction solution of the Mie theory is equivalent to a simplified form of the parabolic wave equation known as the eikonal approximation in light scattering [28]:


[5]

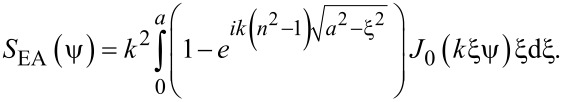



The eikonal approximation S_EA_(ψ) for the parabolic wave equation holds under the same two conditions required for the Mie theory to reduce to the anomalous diffraction theory, namely when the wavelength is small relative to the size of the object, and when the magnitude of the complex refractive index *n* is close to one [28]. Note that the only difference between the anomalous diffraction approximation for the Mie theory (Equation 4) and the eikonal approximation for the PWE (Equation 5) is 2(*n* − 1) vs *n*^2^ − 1 in the exponent of the exponential, and this difference is negligible when *n* ≈ 1 (Supporting Information File 1, Section S5). We also demonstrate this explicitly in Figure S5 in Supporting Information File 1, Section S4, where we calculate S_ADA_(ψ) and S_EA_(ψ) and show that the curves overlap for the 60 nm gold nanospheres examined in this study. Thus, for our sample, the Mie theory is equivalent to the parabolic wave equation, and therefore light propagation through the nanosphere is equivalent in the pc-Mie and the pc-PWE models.

Light propagation in the inc-BL model is a simplified version of that in the preceding two models. The simplification arises when the phase term δ in the refractive index is set to zero and intensities instead of field amplitudes are measured. With δ = 0 then *n* = 1 + *i*β, and the argument in the integral of Equation 4 becomes




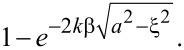




This reflects the loss in the field amplitude passing through the sphere at a distance ξ from its center, and so the loss in intensity is given by the squared amplitude of the exponential term leading to Beer’s law for the absorption *A*(*t*) as a function of the distance 
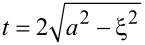
 that the ray travels through the sphere:


[6] $$A\left(t\right)=1-e^{-4k\beta \sqrt{a^{2}-\xi^{2}}}=1-e^{-2k\beta t}.$$

In sum, we have shown that the Mie theory can be simplified for the experimental case considered here, namely a sphere whose refractive index *n* is close to one and whose diameter is much larger than the wavelength λ. Then we show that this simplified form of the Mie theory is essentially equivalent to a simplified form of the parabolic wave equation, which applies under precisely the same conditions, that is, a sphere with *n* ≈ 1 whose diameter is much larger than λ. Thus, the pc-Mie and pc-PWE models provide equivalent procedures for light propagation through the nanosphere. Finally, we show that light propagation in the inc-BL model, namely via Beer’s law, arises from a further simplification of the pc-Mie and pc-PWE procedures in which the phase component of the refractive index is ignored and intensities instead of fields are calculated.

#### 2.3 Propagation of light through the zone plate to the detector plane

Zone plates are diffractive optics with a defined periodic pattern of clear and opaque rings whose transmission function *Z*(*x*′,*y*′) can be written as a cosine series [17]:


[7] $$\begin{array}{l} Z\left(x^{\prime },y^{\prime }\right)=\\ \{\begin{array}{cc} \frac{1}{2}+\frac{2}{\pi }\sum_{m=1,3,...}^{\infty }\frac{1}{m}\cos\left[\frac{m\pi \left({x^{\prime }}^{2}+{y^{\prime }}^{2}\right)}{\lambda f}\right] & {x^{\prime }}^{2}+{y^{\prime }}^{2}\leq r_{\text{zp}}^{\;2}\\ 0\; & {x^{\prime }}^{2}+{y^{\prime }}^{2}>r_{\text{zp}}^{\;2} \end{array}, \end{array}$$

where *r*_zp_ is the radius of the zone plate, which defines its NA. Expanding the cosine function in Equation 7 in terms of complex exponentials yields:


[8] $$Z\left(x^{\prime },y^{\prime }\right)=\frac{1}{2}+\frac{1}{\pi }\sum_{m=1,3,...}^{\infty }\frac{1}{m}\left(\begin{array}{l} \exp\left[\frac{im\pi \left({x^{\prime }}^{2}+{y^{\prime }}^{2}\right)}{\lambda f}\right]+\\ \exp\left[\frac{-im\pi \left({x^{\prime }}^{2}+{y^{\prime }}^{2}\right)}{\lambda f}\right] \end{array}\right).$$

The odd integers *m* correspond to the different diffraction orders of the zone plate, each producing a focus with focal length *f_m_* = *f*/*m*, where *f* is the focal length of the zone plate’s first diffraction order. The intensities of these different foci therefore go as 1/(*m*π)^2^.

Most TXMs operate in first order by using a hollow-cone illumination to remove the zero-order light and by positioning the camera to detect the image produced by the first-order focus. Setting *m* = 1 in Equation 8 yields two exponential terms corresponding, respectively, to the first-order divergent and convergent foci of the zone plate. The convergent term exp(−*i*π(*x*′^2^ + *y*′^2^)/λ*f*)) is exactly the standard quadratic phase shift term exp(−*ik*(*x*′^2^ + *y*′^2^)/2*f*)) that arises from a conventional lens, and this is the term we and the other two 3D models have used to approximate the zone plate as a lens.

This is an excellent and widely used approximation [20–21,29] because it has been shown that zone plates with a sufficient number of zones (>200) yield diffraction-limited Airy disk patterns at each focus *f_m_* = *f*_1_/*m* [30–31]. Indeed, we have shown by numerical computation that the diffraction pattern produced by the zone plate used in this study (900 zones, with outermost zone width *dr*_n_ = 25 nm) quantitatively agrees with the expected Airy disk pattern for the lens with the equivalent NA [32].

#### 2.4 pc-Mie model equations

Combining the preceding steps (2.1–2.3) for our model leads to equations for both the plane wave and the scattered wave fields at the camera:


[9]

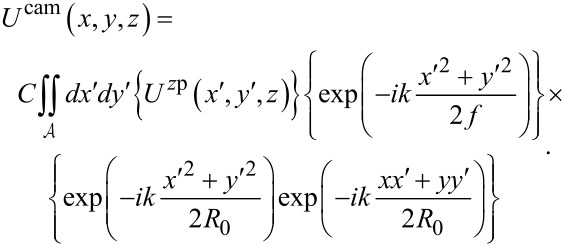



Note that *x* and *y* refer to camera coordinates here, while the variable *z* refers to the *z* shifts of the nanosphere along the optical axis relative to the microscope’s true focus position *R*_f_ (see Figure 1b for definitions of the variables used). The three bracketed terms in Equation 9 correspond to the three model components: (1) the field *U*^zp^(*x*′,*y*′) arriving at the zone plate for either the plane wave or the scattered wave, (2) the approximation of the zone plate as a lens, which yields a quadratic phase shift with its focal length *f* corresponding to the zone plate’s first-order focus, and (3) Fresnel diffraction through the zone-plate aperture, which also gives rise to the constant *C* in front of the integral. 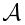 is the circular aperture of the zone plate over which the double integration is performed.

As derived in Supporting Information File 1, Section S3, the fields arising at the zone plate *U*^zp^(*x*′,*y*′) for the plane and scattered waves are:


[10]

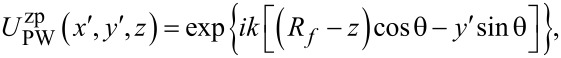




[11] $$U_{\text{SW}}^{\text{zp}}\left(x^{\prime },y^{\prime },z\right)=\frac{S_{\theta }\left(x^{\prime },y^{\prime }\right)}{-ik}\left(\frac{\exp\left(ikR_{\text{M}}\left(z\right)\right)}{R_{\text{M}}\left(z\right)}\right),$$

where θ corresponds to the polar angle of illumination and *R*_M_ is the distance from the nanosphere to a point (*x*′,*y*′) on the zone plate.

Substitution of Equation 10 into Equation 9 leads to the final expression for the plane-wave field at the camera, which depends on the camera coordinates (*x*, *y*) and the amount of defocus *z* of the sample:


[12]

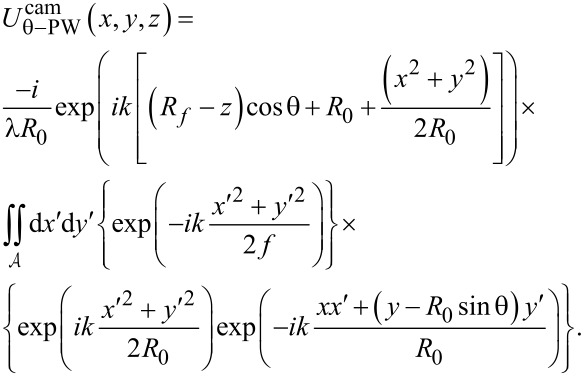



Similarly, substitution of Equation 11 into Equation 9 leads to the final expression for the scattered-wave field at the camera, but as shown in Supporting Information File 1, Section S6, this expression can be simplified by a paraxial approximation. This leads to a solution that takes a standard form [33], namely the Fourier transform of the lens pupil function (in brackets below) computed over the lens circular aperture 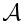:


[13]

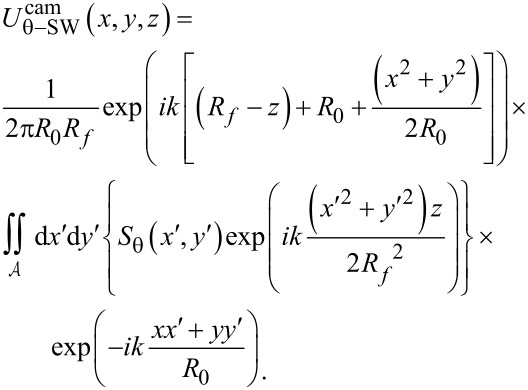



Note that the pupil function is given by the product of a term accounting for the scattered field in the zone-plate plane, *S*_θ_(*x*′,*y*′), and a term accounting for the defocus wavefront aberration,


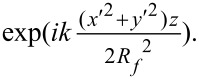


The expressions in Equation 12 and Equation 13 for the plane-wave and scattered-wave fields at the camera are then added to produce the total field at the camera:


[14] $$U_{\theta }^{\text{cam}}=U_{\theta -\text{PW}}^{\text{cam}}+U_{\theta -\text{SW}}^{\text{cam}}.$$

Note that the constant terms in front of the plane and scattered wave solutions are different in Equation 12 and Equation 13 above, and so these must be incorporated in Equation 14 to account for the proper proportions of each in the sum. As indicated by the subscript θ, the field at the camera 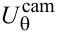 depends on the oblique angle of plane wave illumination (the polar angle θ) along the ellipsoidal mirror’s long axis.

#### 2.5 inc-BL model equations

The inc-BL model has been previously described [20]. Briefly, the model assumes incoherent illumination such that the amount of light absorption at any point in the nanosphere is convolved with the point-spread function *h*(*r*, *z*) of the objective. As in both our pc-Mie model and the pc-PWE model, the objective zone plate is also approximated by a lens. Given the radial symmetry of the nanosphere, the inc-BL model for the intensity *I*^cam^ at the camera can be written as:


[15] $$I^{\text{cam}}\text{​}\left(Mr\right)=1-\text{​}\int_{-a}^{a}\left(\mu \left(r\text{​},z\right)\exp\left(-\text{​}\int_{-a}^{z}\mu \left(r\text{​},\xi \right)\text{d}\xi \right)\right){⊗}_{r}h\left(r\text{​},z\right)\text{d}z,$$

where (*r*, *z*) are cylindrical coordinates, *M* the microscope magnification, and µ the linear absorption coefficient defined for a nanosphere of radius *a* by:


[16] $$\mu \left(r,\xi \right)=\{\begin{array}{cc} 2k\beta & r^{2}+\xi^{2}\leq a^{2}\\ 0\; & r^{2}+\xi^{2}>a^{2} \end{array}.$$

Note that within the gold nanosphere, the linear absorption coefficient µ(*r*, *z*) is a constant and moves outside of the integral of Equation 15. The remaining exponential term in the integral reflects the accumulated absorption at different depths in the nanosphere defined by Beer’s law (Equation 6), which is then convolved with the point-spread function *h*(*r*, *z*) of a lens (whose NA equals that of the zone plate). Supporting Information File 1, Section S7, provides more details about the model and its implementation.

### 3 Comparisons of the models to each other and to experimental data

#### 3.1 Experimental measurements

**Figure 2:**
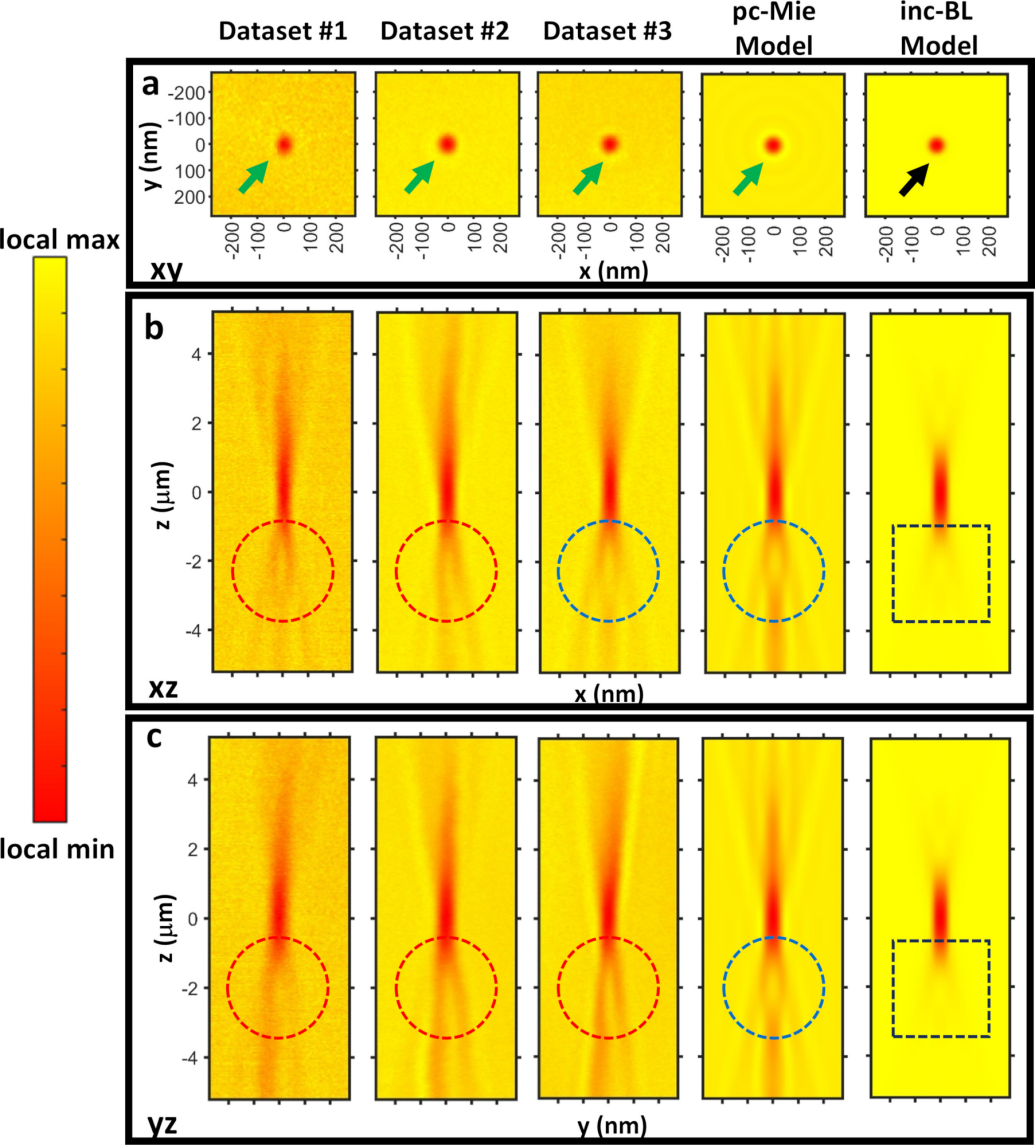
The pc-Mie model is more similar to the experimental data than the inc-BL model. Shown are *xy* (a), *xz* (b) and *yz* (c) views of the experimental data from three different 60 nm gold nanospheres collected months apart (Datasets #1, #2, and #3), and compared to the model predictions for a 63.2 nm nanosphere, which is the diameter of the nanosphere in Dataset #2 as measured by SEM. Exposure times were 2 s (Dataset #1), 8 s (Dataset #3), and an average of 3 × 14 s exposures (Dataset #2). Green arrows point to a contrast reversal around the nanosphere seen in both the data and the pc-Mie model, but absent in the inc-BL model (black arrow). An axial contrast reversal is also present in both the data and the pc-Mie model (dashed circles), but absent in the inc-BL model (dashed square). The red dashed circles highlight the lateral asymmetry in the axial images mostly characteristic of the experimental data, while the blue dashed circles highlight the lateral symmetry characteristic of the axial images from the models.

To test the models against real data, we collected a series of 2D images of 60 nm gold nanospheres at different amounts of defocus to generate a 3D dataset. Thus *z* values correspond to the shift of the microscope stage from its true focus location. We used the Helmholtz-Zentrum Berlin TXM operating at 510 eV at the electron storage ring BESSY II. To assess the reproducibility of the image data, we initially collected nine different 3D datasets under similar imaging conditions, but under different adjustments of the beamline due to fluctuations of the beam. The detailed data collection and pre-processing workflow is summarized in the Experimental section, and includes alignment of the focal-plane series to correct for stage jitter using larger 270 nm gold shells that were also included in the sample, plus correction for temporal fluctuations in the X-ray light source occuring during collection of the focal-plane series. Other than these basic corrections for jitter and source flicker, the data were not subjected to any other processing or reconsturction procedures. Three representative final 3D images are shown below in Figure 2.

#### 3.2 Model comparisons: the effects of oblique illumination and the phase component of the refractive index

We compared the experimental data to the model predictions (Figure 2). We used Equations 12–14 above for the pc-Mie model to compute the predicted partially coherent image of the gold nanosphere under the condenser illumination conditions used in the pc-PWE model. Specifically, we assumed that the sample was illuminated by oblique plane waves at the mean polar angle of θ_m_ = 1.01° and that the coherence patch size around the circumference of the ellipsoidal mirror was small, *p*_φ_ = 1°. To do this, we calculated the field 
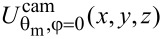
 and then rotated it through 360° by 1° increments. Then we computed intensities at each angle and added them to yield the final image. Note that under these conditions, as described above (Table 1), the pc-Mie and pc-PWE models are equivalent, but in this study we have used our implementation of the pc-Mie model to compute predicted images, and so use this as the label for all images. For the inc-BL model, we used Equation 15 and Equation 16.

We found that the images from the pc-Mie model (Figure 2) were qualitatively similar to the experimental data in many respects: (1) After normalization, the images from both the model and data spanned similar intensity ranges; (2) the complex structure of the *xz* and *yz* images were similar showing a central in-focus region with high absorption that gave rise to a double-cone pattern of out-of-focus light above and below focus; (3) a contrast reversal occurred below focus in both experiment and theory, whereas no such reversal occurred above focus, and instead the absorption at the center of the bead slowly decayed; and (4) a contrast reversal also occurred around the nanosphere perimeter in the in-focus image.

As noted before, the pc-Mie model predictions (Figure 2) were calculated using a small azimuthal coherence patch size of 1°. We found that larger coherence patch sizes led to increasing deviations between the model and the experimental data. Specifically, as this azimuthal angular patch size increased, the increased coherence in the model led to more and more ringing in the predicted images (Supporting Information File 1, Section S8). Our observation is consistent with the pc-PWE model, which also used a small azimuthal coherence patch size in comparing their model to a phantom test sample [21].

The inc-BL model showed significantly poorer agreement with the experimental data. While this model (Figure 2) also yielded an absorbing nanosphere image in *xy*, it did not account for either the axial or in-focus contrast reversals nor the extended axial elongation of the measured image, and also only faintly showed the pronounced double cone pattern of out-of-focus light present in the measured image. Thus, the pc-Mie model with a small coherence patch delivered significantly more accurate predictions than the inc-BL model.

**Figure 3:**
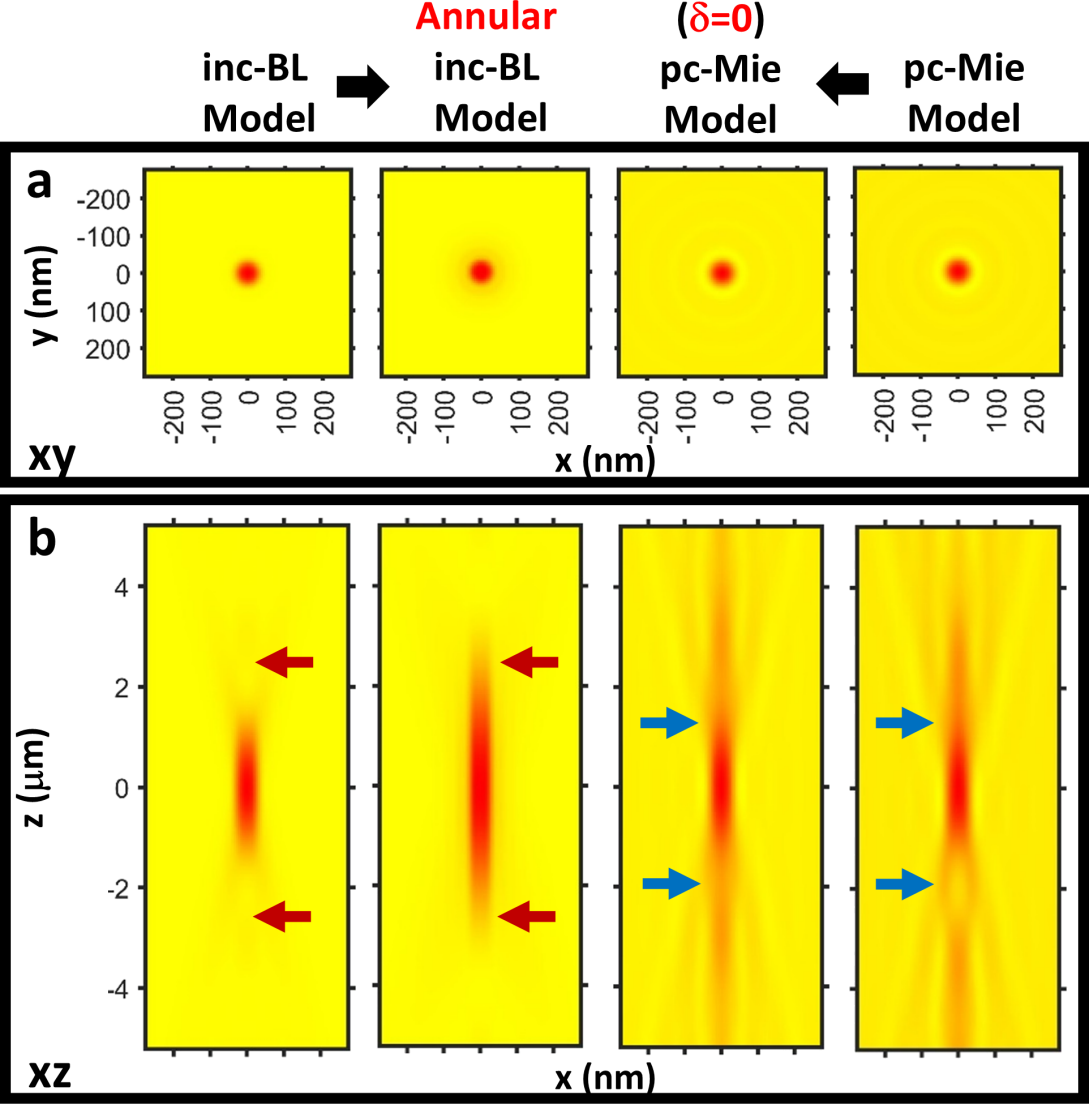
Key differences between the pc-Mie and inc-BL models are the presence or absence of annular illumination and the phase term δ in the refractive index. *xy* (a) and *xz* (b) images of the nanosphere are shown. Simulating annular illumination in the inc-BL model introduces an axial elongation (red arrows, b). Setting δ = 0 in the pc-Mie model eliminates the axial asymmetry (blue arrows). Thus, much of the basic structure of the inc-BL image can be explained by the lack of annular illumination and neglect of the phase term δ in the inc-BL model.

To understand how these different model predictions arose, we evaluated the effect of the differing assumptions made by the models. The inc-BL model presumes incoherent illumination, implying illumination over the full angular range of the zone-plate NA, whereas the pc-Mie model accounts for the mean-angle annular illumination present in the real microscope. To simulate this annular illumination effect in the inc-BL model, we changed the model’s microscope PSF to introduce annular collection of light via an apodized objective lens (Supporting Information File 1, Section S7). We found that this led to an axial elongation of the nanosphere image in the inc-BL model (Figure 3), suggesting that oblique, annular illumination contributed to the more elongated images in the pc-Mie model.

Another key difference between the pc-Mie model and the inc-BL model is that the refractive index of the pc-Mie model includes both absorption and phase terms, whereas the inc-BL model neglects the phase term. To simulate this situation, we set δ = 0 in the pc-Mie model and found that this eliminated the axial contrast reversal (Figure 3). This demonstrates that this contrast reversal arises from the phase properties of the sample and underscores the importance of including this term in the model to accurately account for the measured data.

#### 3.3 Introduction of condenser tilt into the pc-Mie model accounts for the lateral asymmetries in the data

**Figure 4:**
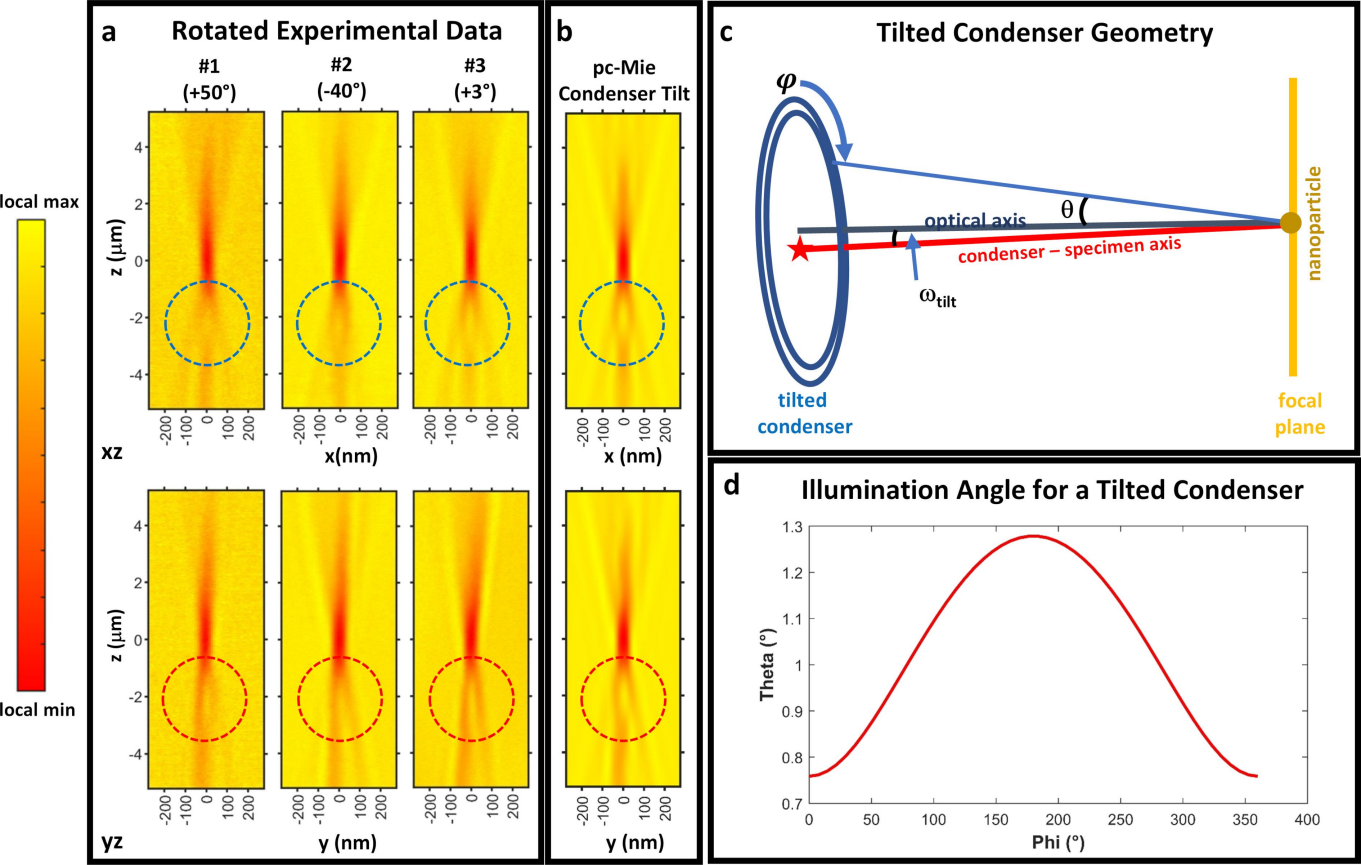
Rotation of the experimental data (a) significantly increased both their similarity to each other and to the predictions of the pc-Mie model (b). Each experimental dataset was rotated around the optical axis by the amount indicated in parentheses (a, see also Figure S7 in Supporting Information File 1, Section S9). Note that rotation of the data eliminated the axial asymmetry in the *xz* views (a, blue dotted circles), and enhanced it in the *yz* views (a, red dotted circles). To account for this *yz* asymmetry, we modified the pc-Mie model by tilting the incoming beam–condenser axis by ω_tilt_ = 0.26° relative to the zone plate–camera axis (c), where ω_tilt_ was determined by estimating the tilt from the *yz* views of the experimental data (Supporting Information File 1, Section S10). This led to a dependence of the polar illumination angle θ on the azimuthal angle φ (d), which was used to produce the predicted images (b), which now more closely resembled the experimental data.

While the pc-Mie model accounted reasonably well for many qualitative features of the experimental data, it failed to predict a lateral asymmetry that we consistently observed (highlighted in Figure 2 with the red and blue circles) and that varied from day to day in the data. We found we could eliminate the day-to-day variations by rotating each dataset around the optical axis by an appropriate angle (Supporting Information File 1, Section S9). Following this rotation, the experimental data resembled each other and also the model, except that the data were slightly tilted in *yz* views compared to the model (Figure 4a vs Figure 2).

We obtained a rough estimate of this tilt in the data by drawing a line along the apparent tilt axis and measuring its angle with the *z* axis, which we found was 0.26° (Supporting Information File 1, Section S10). This tilt could arise from a misalignment of some optical component, for example, a tilt of the incoming beam relative to the optical axis defined by the zone plate and camera (Figure 4c). In this case, the condenser will be aligned relative to the tilted incoming beam and will lead to polar angles θ of plane wave illumination that vary as a function of the azimuthal angle φ around the ellipsoidal mirror circumference (Figure 4d). This dependence θ = θ(φ) can be calculated based on the tilt angle ω_tilt_ = 0.26° and the geometry of the ellipsoidal mirror (see Supporting Information File 1, Section S11, for the equation and its derivation). We used this dependence θ = θ(φ) for a tilted condenser to calculate the image arising from such a tilt by adding the intensities at 1° increments of the azimuthal angle φ using the field 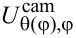 for each φ.

We found that such a tilt in the condenser (Figure 4) yielded predicted nanosphere images that were in good qualitative agreement with the lateral asymmetries observed in the experimental images of the nanosphere. This analysis therefore suggests a misalignment of ≈0.26°, which might arise for example from a tilt in the incoming beam relative to the zone plate–camera axis.

#### 3.4 Quantitative comparisons of radial intensity profiles reveal a discrepancy between the pc-Mie model and the data

We next evaluated the quantitative accuracy of the pc-Mie model by computing radial intensity profiles through the center of the nanosphere images. The nanosphere center was defined as the minimum-intensity pixel in the 3D dataset. The focal plane containing this minimum-intensity pixel was defined as best focus. We then computed an average radial intensity in this best focus plane. For this analysis, we used the non-tilted condenser model because its in-focus radial average was the same as that of the tilted condenser model (Supporting Information File 1, Section S12) and the non-tilted condenser model was much easier to compute. To enable a more precise quantitative analysis, we first extensively characterized the gold nanospheres by both scanning and transmission electron microscopy (SEM and TEM, Supporting Information File 1, Section S13) to determine the nanosphere’s shape, composition, diameter, and density.

Both SEM and TEM showed that the nanospheres were indeed roughly spherical and of similar size (Supporting Information File 1, Section S13). Specifically, we measured the diameter of 35 nanospheres by SEM and obtained a value of 62.2 ± 2.9 nm. Furthermore, TEM images showed that the nanospheres were solid and had roughly similar densities throughout. By TEM, the interior of the nanospheres did exhibit some lightly shaded stripes, characteristic of a channeling effect expected to arise from a crystalline structure. To investigate this further, we performed selected area electron diffraction on the nanospheres, which revealed the presence of crystalline domains in the nanospheres with the lattice spacing of gold (Supporting Information File 1, Section S13, Figure S12a). Furthermore, a high-resolution TEM image showed the crystalline order extended over the full particle. Fourier transform of a sub-region of this image yielded a lattice spacing for Au(111) of 0.236 nm, close to the nominal value of 0.235 nm (Supporting Information File 1, Section S13, Figure S12b). Finally, an energy-dispersive X-ray spectroscopy analysis revealed that the principal element within the nanosphere was gold with very small contributions from carbon, oxygen, and potassium (Supporting Information File 1, Section S13, Figure S11d). We conclude the nanospheres are solid, nearly spherical and predominantly composed of crystalline gold, which enables us to accurately calculate their expected radial intensity profiles and absorptions.

**Figure 5:**
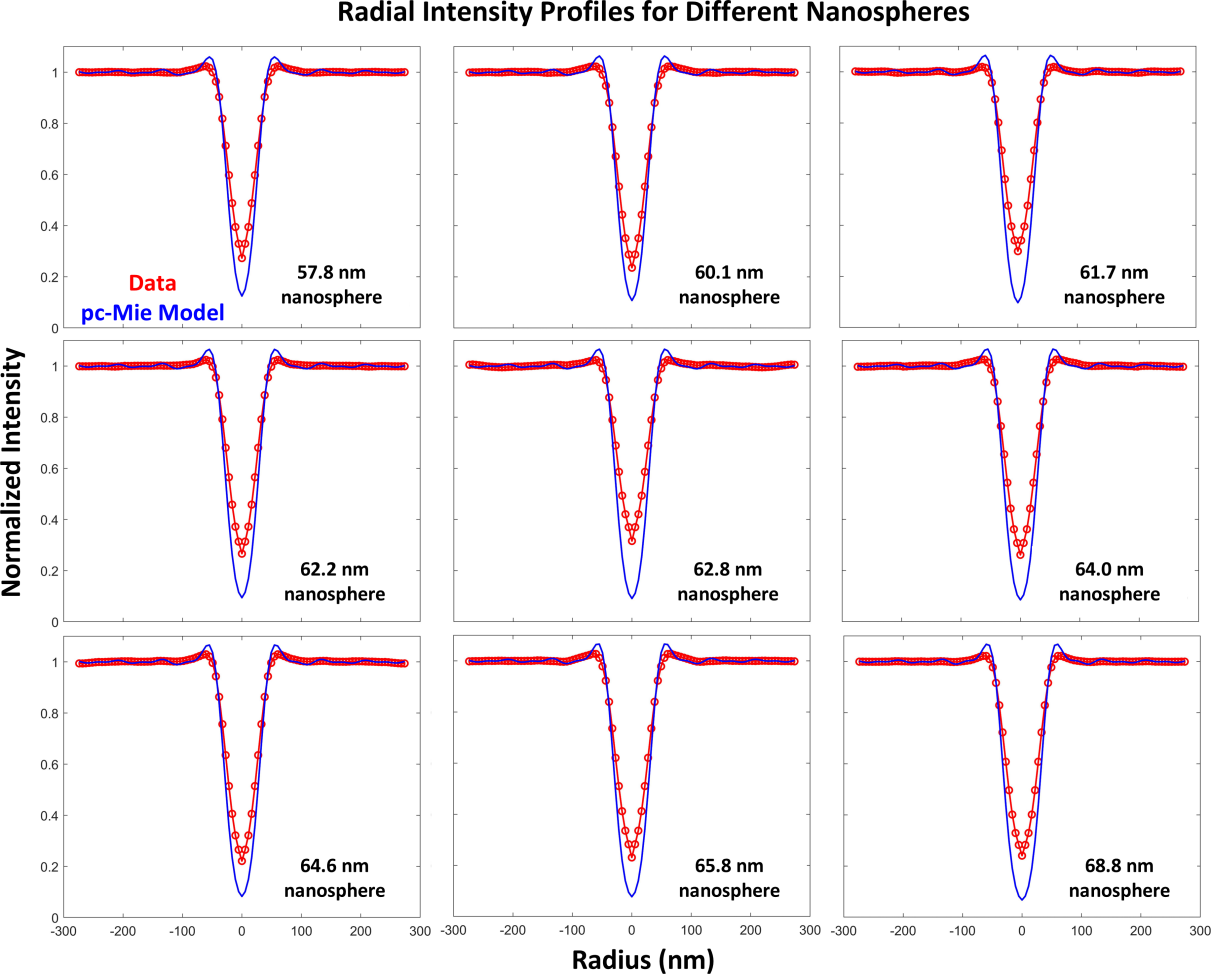
Comparison of measured and predicted radial intensity profiles for nine different nanospheres whose diameters were measured by SEM. An overshoot and undershoot of the pc-Mie model compared to the data are seen in all cases.

We then collected 3D TXM images of nine different nanospheres, each of which had been pre-imaged by SEM to determine its diameter. Based on the SEM images, these nine nanospheres were selected to exclude the outlier sizes and shapes that we had also found by TEM. The 3D TXM images thereby enabled a direct comparison of radial intensity profiles for each nanosphere with those predicted by the model calculated for that specific diameter and the density of crystalline gold. For all nine nanospheres we observed a similar significant undershoot and a slight overshoot of the pc-Mie model compared to the data (Figure 5).

This significant undershoot cannot be explained by errors in estimating the diameters of the slightly oblong nanospheres by SEM, as these estimation errors lead to only small differences in the predicted radial intensity profiles (Supporting Information File 1, Section S14). The same is true for an error in estimation of the gold density, where we found that to match the data the nanosphere would require a nanosphere density of 60% of that for crystalline gold (Supporting Information File 1, Section S14), a result completely inconsistent with the TEM analysis of the nanospheres.

#### 3.5 The microscope underestimates the absorption of the 60 nm gold nanospheres

To understand the consequences of the quantitative discrepancy in the radial profiles between the model and the data, we calculated the absorption of each nanosphere, since this is a commonly measured parameter in samples imaged by TXMs. An exact measurement of experimental absorption is non-trivial because the background around the nanosphere varies slowly with distance from the nanosphere center. Therefore, to achieve a robust estimate of absorption, we integrated the experimental radial intensity profile starting at different points beyond the central inverted peak of the nanosphere. The absorption as a function of the integration endpoint is given by:


[17] $$A\left(r_{\text{end}}\right)=\int_{0}^{2\pi }\text{d}\theta \int_{0}^{r_{\text{end}}}\left(1-I\left(r\right)\right)r\text{d}r=2\pi \int_{0}^{r_{\text{end}}}\left(1-I\left(r\right)\right)r\text{d}r,$$

where *I*(*r*) is the radial intensity profile and *r*_end_ is the endpoint of the integration, which we increased stepwise to include more and more of the tails of the intensity profile. This yielded multiple absorption estimates from a single nanosphere’s best-focus image, enabling an estimate of the mean absorption and its standard deviation.

We performed an analogous measurement of absoprtion from best-focus images produced by the pc-Mie model, and finally we also calculated the absorption predicted by Beer’s law:


[18] $$A_{\text{BL}}=2\pi \int_{0}^{a}\left(1-\exp\left(-4k\beta \sqrt{a^{2}-\xi^{2}}\right)\right)\xi \text{d}\xi ,$$

where ξ is the radial coordinate in the nanosphere and *a* is the radius of the nanosphere as measured by SEM.

**Figure 6:**
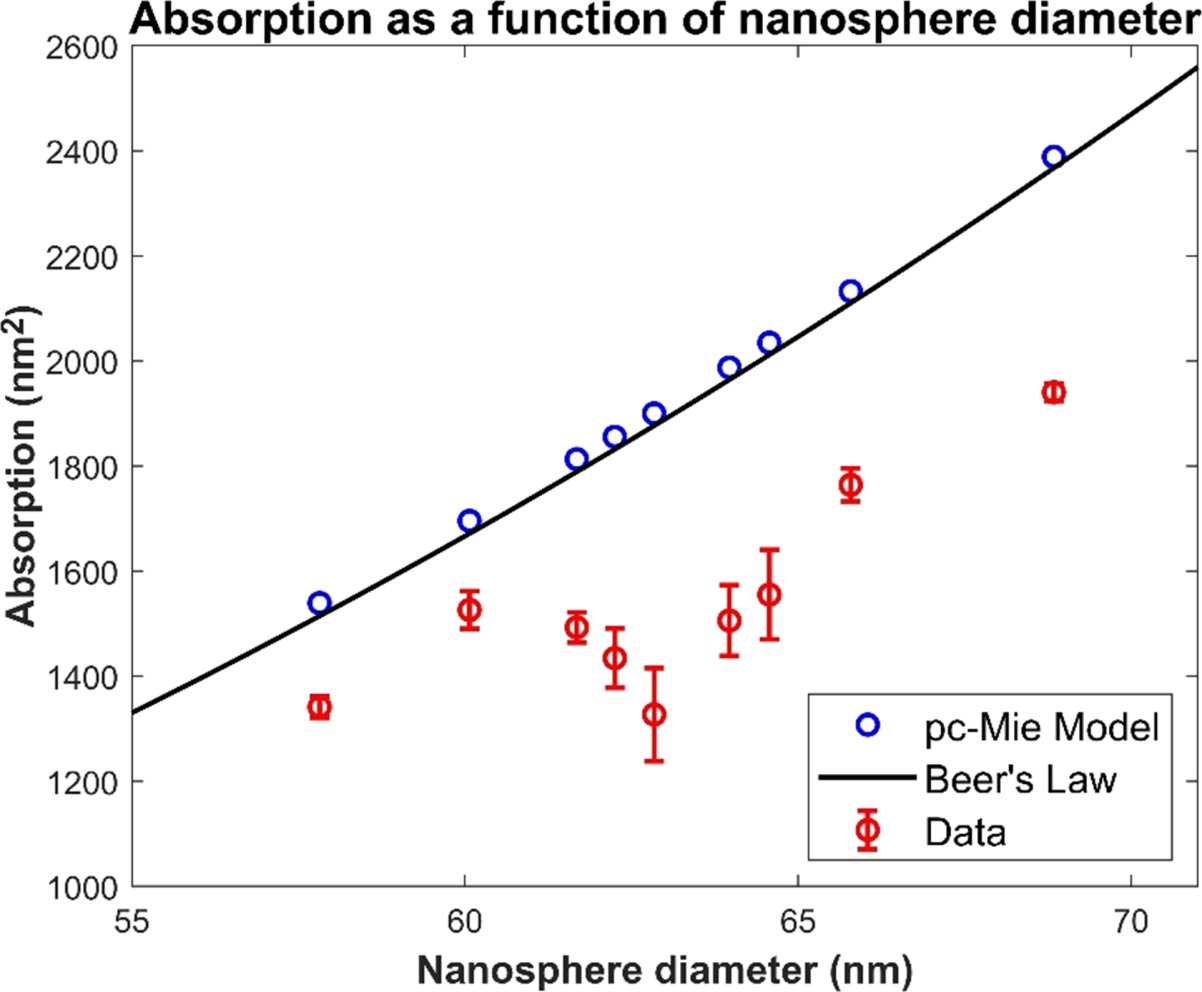
Absorptions from the nine nanosphere images are significantly less than predicted by either the pc-Mie model or Beer’s law. This suggests that the microscope may reduce the actual absorption due to some as yet unidentified feature that must ultimately be incorporated into the model.

We found that the predicted absorption from the pc-Mie model was nearly identical to Beer’s law (Figure 6). However, consistent with the radial profiles in Figure 5, we found that the measured absorption from each nanosphere was always significantly less than that predicted by either the model or Beer’s law. This discrepancy between Beer’s law and the measured data was much larger than the standard deviation of the data. As described above, the discrepancy between the model and the data cannot be attributed to uncertainties about the nanosphere geometry. We conclude therefore that the microscope is likely to significantly underestimate the nanosphere’s absorption, and so our results suggest that there is some feature of the actual microscope not incorporated into the model that reduces the measured nanosphere absorption.

## Discussion

We have developed a 3D soft TXM model and compared it to the two most recent soft TXM models, both of which also incorporated 3D imaging on the microscope (Table 1). We showed that, for the case of the gold nanosphere examined here, the simplest form of our pc-Mie model was essentially identical to the pc-PWE model [21]. This is because we showed that Mie theory and the parabolic wave equation yield virtually identical predictions for soft X-ray light propagation through a 60 nm gold nanosphere.

Our pc-Mie model however yielded predictions very different from the other 3D TXM model, namely the inc-BL model [20]. A key difference was that our model included the phase component δ of the sample’s refractive index, and this led to predicted axial images with a marked contrast reversal consistent with the experimental data. By comparison, when we set δ = 0 in our pc-Mie model, the predicted axial images were completely symmetric, as also predicted by the inc-BL model, where the phase component is also not incorporated. This result demonstrates that phase must be included in any soft TXM model for an accurate description of 3D imaging. This is significant because both spectromicroscopy and the most commonly used tomographic reconstruction procedures for TXMs ignore the phase term in the refractive index and presume instead that the image arises solely from absorption, namely Beer’s law. Thus, future work should focus on exactly how the phase term δ influences these different imaging applications to enable more accurate measurements.

We identified both qualitative and quantitative discrepancies between our model and the measured data from 60 nm gold nanospheres. Our qualitative comparisons revealed a lateral asymmetry consistently present in the measured data that was absent in the model. We could account for this lateral asymmetry by introducing a small tilt (0.26°) between two axes, the first defined by incoming beam and condenser and the second by zone plate and camera, but other small misalignments in the microscope’s optical train could also play a role. In itself, the presence of a small tilt is not surprising given the difficulty in precisely aligning all optical components. Importantly, however, we consistently observed a similar amount of tilt in all datasets collected, and therefore an accurate model for tomographic reconstruction should also incorporate this tilt in order to achieve optimal 3D reconstructions.

Beyond this qualitative difference due to a slight misalignment in the actual microscope, our quantitative comparison of the model’s predictions to the measured data also revealed a surprising discrepancy: The model consistently overestimated the amount of absorption in the real data. Strikingly, however, the model’s predictions agreed with Beer’s law whereas the measured data did not. This discrepancy did not appear to be due to uncertainties in the geometry of the nanospheres examined as both SEM and TEM analysis of the nanospheres indicated a reasonably well-defined spherical geometry composed of crystalline gold. These results suggest that the microscope imaging process itself may introduce some additional component such that measured absorptions are 10–20% lower than predicted by Beer’s law.

Our model suggests that this discrepancy might arise because the theoretical data exhibited significantly larger overshoots and undershoots compared to the experimental data. One possible explanation for this is that there is a small background in the microscope images that is currently not accounted for in the model. Such a background would reduce both the over and undershoots in the measured data, but other explanations are also possible. Therefore, it will be important in future work to identify exactly what feature(s) of the actual imaging process introduce this absorption discrepancy, such that it can be corrected.

This goal can be achieved by extending our current pc-Mie model to account more accurately for all of the features in the actual microscope (Table 1). This should include incorporation of (1) the zone plate and its accompanying non-ideal features such as struts [34], rather than the approximation as an ideal lens; (2) partial coherence that is based on future measurements of the microscope’s coherence patch size and incorporates the full angular range of illumination rather than just the mean angle; and (3) the spiral scanning of the condenser focal spot rather than a stationary condenser. It is likely that at least one of these three features is responsible for the quantitative discrepancies that we have measured here between the data and the model. Identifying the source of this discrepancy will be important not only for producing an accurate imaging model for improved reconstruction procedures but also for ensuring accurate quantitative measurements with the microscope.

Future work should also explore how our model could be extended from soft to either tender or hard X-rays. At these higher energies, both the Mie theory for light propagation through a sphere and the lens model for the zone plate should still be valid. However, with increasing energy, the ratio of δ/β progressively increases, indicating that phase effects increasingly dominate. With hard X-rays, phase effects are so dominant that Zernike phase contrast is required, and so our model would have to be extended to account for this imaging modality. With tender X-rays, there is still some absorption if the nanosphere is large enough. However, given a larger object, our simple mean-angle model for the condenser might no longer be valid, and a more general model accounting for the full angular range of condenser illumination is likely to be required. Thus, further comparisons between theory and experiment will be needed to properly extend our model to tender X-rays, and the model itself must be extended to phase contrast imaging for hard X-rays.

## Conclusion

We have developed an experimental and theoretical framework to evaluate transmission X-ray microscope models. We find that a relatively simple model of the microscope yields very good qualitative agreement with experimental data, which can be further improved by incorporating a small tilt between microscope components. To achieve this reasonable qualitative agreement with the data, we found that the model must account for both β and δ of the sample’s complex refractive index. However, we also showed that this simple model has a deficiency: It does not agree quantitatively with the measured data because the data exhibit less absorption than predicted by Beer’s law. Achieving quantitative agreement will require first identifying why the microscope underestimates absorption and then incorporating this feature(s) into the model. This will then enable more quantitatively accurate measurements in both spectromicroscopy and biomedical imaging.

## Experimental

**Figure 7:**
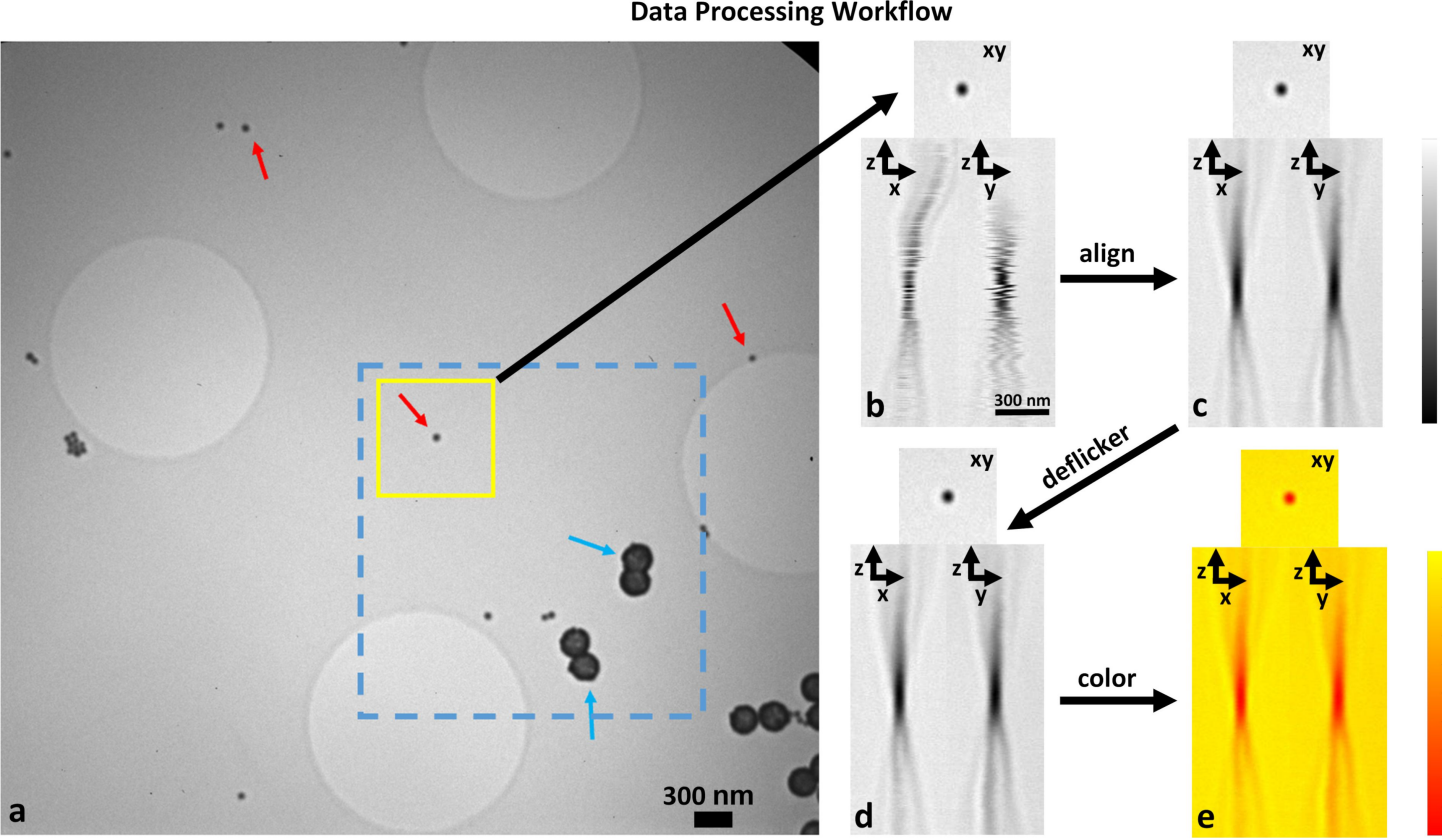
Data processing workflow. A full-field in-focus image is shown from the 3D image stack. Large circles are holes in the carbon foil of the grid. Red arrows point to selected 60 nm gold nanospheres and blue arrows to selected 270 nm gold shells (containing a silicon core). A sub-image (yellow box) around an isolated 60 nm gold nanosphere was extracted to produce a 3D volume, whose *xy*, *xz*, and *yz* cuts are shown (b). Stage jitter and drift were corrected (c) using the StackReg plugin in imagej applied to the 3D stack defined by the dotted blue rectangle in (a), which includes several of the 270 nm gold shells that are essential for good alignment of the stack. Plane-to-plane variation in the X-ray illumination (flicker) was corrected by normalizing the sum of intensities in each plane to a constant (d). A false-color scale (“autumn” in Matlab) was used to better visualize the intensity range, with red corresponding to regions of highest absorption and yellow to regions of highest transmission (e). Scale bar = 300 nm.

The measured data [35] were obtained by allowing both 60 nm gold nanospheres and 270 nm gold shells from a colloidal gold suspension to dry onto holey carbon-coated gold finder grids (Quantifoil). 3D volumes from selected regions of the grid were then collected using the Helmholtz-Zentrum Berlin TXM operating at 510 eV at the electron storage ring BESSY II. A 25 nm zone plate was used with the microscope configured for a magnification of ≈3600×. This led to a pixel size in each 2D image of 5.5 nm. A 3D volume was generated by collecting 301 2D images that were separated from each other in *z* by 40 nm, thereby yielding a final voxel size for the 3D volume of 5.5 nm × 5.5 nm × 40 nm. Finally, these raw 3D images of the 60 nm nanospheres were processed to reduce stage jitter and flickering of the X-ray light source (Figure 7). Supporting Information File 1 contains additional information for all other experimental procedures and analyses.

Matlab was used to compute all model predictions. Here we summarize the computational approach for calculating the pc-Mie model. Supporting Information File 1 contains detailed descriptions for all other model calculations. We validated the code for computing the fields 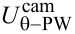 and 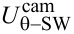 for the pc-Mie model Equations 12 and 13 by comparing results for several approaches including numerical integration by the trapezoidal rule, numerical integration by steepest descent contour deformation designed for highly oscillatory integrals (a custom version of the PathFinder code [36]), and, in the case of the plane-wave field, using a change of variables to convert Equation 12 into Lommel’s integral, which can be solved by a sum of Lommel functions [37]. With vectorized Matlab code, these integrals could be computed in less than 5 min using an Intel core i9 processor. All code is available upon request.

## Supporting Information

The Supplement is a single document with multiple sections listed according to their order of citation in the main text. The different sections provide (1) detailed explanations of the different models: S1 – Mean angle condenser; S2 – Plane-wave model of the condenser; S3 – Extension of the Mie theory to oblique plane waves; S4 – Simplification of the Mie theory to the anomalous diffraction approximation; S5 – Equivalency of the Mie theory and the parabolic wave equation; S6 – Calculation of the nanosphere’s plane-wave and scattered-wave fields at the camera; S7 – Incoherent Beer’s law model; S11 – Calculation of polar tilt angle as a function of azimuthal angle in a tilted condenser; (2) calculation of specific model predictions: S8 – Effect of coherence patch size in the mean-angle condenser model; S12 – Radial intensity profiles of the tilted vs. non-tilted theory; S14 – Sensitivity of radial intensity profiles to deviations in nanosphere geometry; and (3) descriptions of experimental procedures and measurements: S9 – Rotation of the experimental data around the optical axis; S10 – Estimation of the axial tilt in the data; S13 – Structural characterization of the 60 nm gold nanospheres by SEM and TEM.

File 1 Supplement.

## Data Availability

Data generated and analyzed during this study is openly available in HZB Data Service at https://doi.org/10.5442/ND000011.
