# Supplementary material for: Towards a quantitative theory for transmission X-ray microscopy
Source: Beilstein J Nanotechnol. 2025 Jul 15;16:1113–28. doi: 10.3762/bjnano.16.82 (PMC12278111; doi:10.3762/bjnano.16.82)
Supplement: File 1 — Supplement. [file Beilstein_J_Nanotechnol-16-1113-s001.pdf]

BEILSTEIN JOURNAL OF NANOTECHNOLOGY

Supporting Information

for

Towards a quantitative theory for transmission X-ray microscopy

James G. McNally, Christoph Pratsch, Stephan Werner, Stefan Rehbein, Andrew Gibbs, Jihao Wang, Thomas Lunkenbein, Peter Guttmann and Gerd Schneider

Beilstein J. Nanotechnol. **2025,** *16,* 1113–1128. doi:10.3762/bjnano.16.82

Supplement

License and Terms: This is a supporting information file under the terms of the Creative Commons Attribution License (https://creativecommons.org/licenses/by/4.0). Please note that the reuse, redistribution and reproduction in particular requires that the author(s) and source are credited and that individual graphics may be subject to special legal provisions.
The license is subject to the *Beilstein Journal of Nanotechnology* terms and conditions: (https://www.beilstein-journals.org/bjnano/terms)

S1. Mean-angle condenser

The X-ray microscope condenser is a truncated glass capillary that acts as an ellipsoidal mirror. The X-ray source is situated at the left focus of the ellipse (the entrance focus) and the sample is situated at the right focus (the exit focus). A central aperture stop blocks zero-order illumination of the zone plate and leads to illumination of the sample over an annular range 0.83–1.18°, as determined by the geometry of the truncated ellipse (Figure S1a).

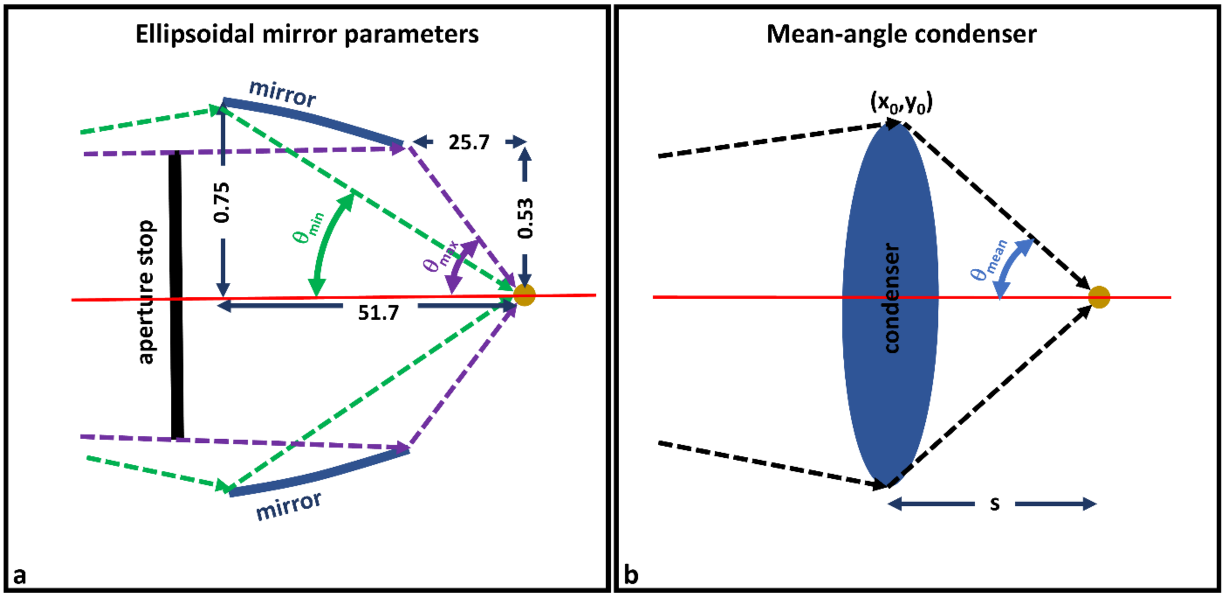

Figure S1: a.) Shown is the ellipsoidal mirror (which is a small segment of the full ellipse) used to focus light onto the sample (gold nanosphere) through an annular range spanning the minimum (green) to maximum (purple) polar angles of illumination (diagram is not to scale). These angles can be determined from the edges of the mirror (numbers shown are mm). $\theta_{max} = arctan(0.53/25.7) = 1.18°$ and $\theta_{min} = arctan(0.75/51.7) = 0.83°$. b.) A simplified model for the ellipsoidal mirror is given by illumination at a single polar angle, $\theta_{mean} = 1.01°$, which is the mean of θ_{max} and θ_{min}.

The pc-PWE and pc-Mie models assume a mean polar angle of illumination (Figure S1b). This mean polar angle is given by the mean of the angular range of the condenser $\theta_{min} = 0.83°$ to $\theta_{max} = 1.18°$, yielding $\theta_{mean} = 1.01°$. The ellipse geometry can then be used to determine the distance s of this mean-angle plane from the ellipsoidal-mirror exit focus and the height y_0 of the ellipsoidal-mirror circumference in this mean-angle plane (Figure S2).

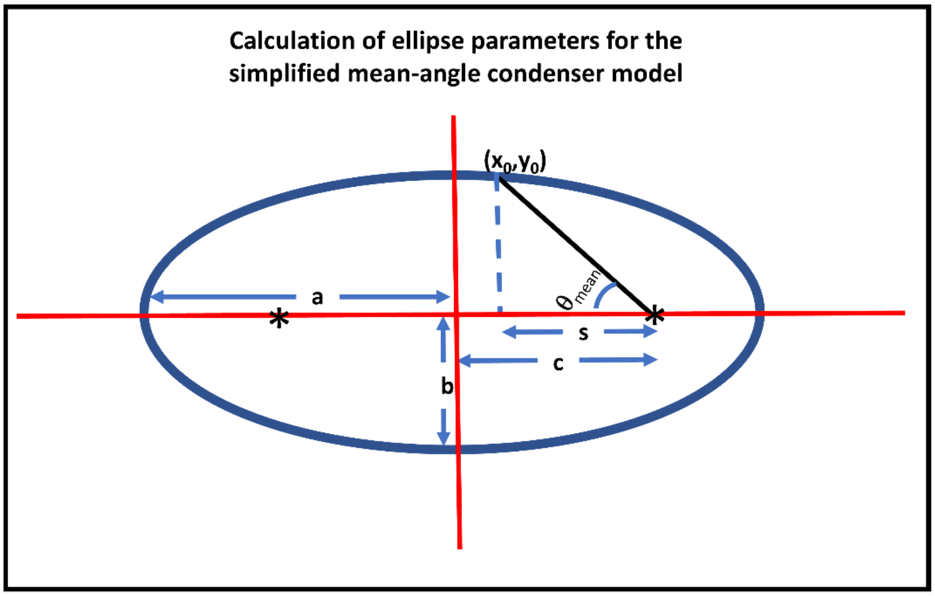

Figure S2: Shown is a cross-section of the prolate ellipsoid from which the truncated ellipsoidal-mirror condenser is derived. The major and minor axes are given by $a = 4875$ mm and $b = 5.16$ mm respectively, and the distance from the center of the ellipse to a focus is given by $c = \sqrt{a^2 - b^2}$. The simplified mean-angle condenser is defined by the ray from the edge of the ellipse at (x_0, y_0) to the ellipse's exit focus which makes an angle $\theta_{mean} = 1.01°$ with the optical axis (See Figure S1b). y_0 therefore corresponds to the radius of the simplified mean-angle condenser. Finally, note that s is the in-focus distance along the optical axis from the mean-angle condenser plane to the exit focus where the sample is located. To define the geometry of this simplfied annular condenser for future calculations we must determine s and y_0. These can be obtained using the fact that the point (x_0, y_0) lies on the ellipse and therefore must satisfy the equation for the ellipse $x^2/a^2 + y^2/b^2 = 1$. Substituting $y_0 = s * tan\theta_{mean}$ and $x_0 = c - s$, leads to a quadratic equation for s whose solution is: $s = \left(-B + \sqrt{B^2 - 4AC}\right)/(2A)$, where $A = b^2 + a^2 tan^2\theta_{mean}$, $B = -2b^2c$, $C = -b^4$. Using the values above for a, b and θ_{mean} leads to $s = 34.87$ mm and $y_0 = 0.62$ mm. The circumference of the condenser in the mean-angle plane is therefore $2\pi y_0$=3896 µm.

S2. Plane-wave model of the condenser

The ellipsoidal mirror condenser can be modeled as a lens that produces a converging spherical wave and then a plane-wave model for this lens can be obtained by calculating the angular spectrum of the field produced by the lens. The focal length for the lens is equal to the distance s along the optical axis between the condenser focal point and the mean condenser plane (corresponding to an illumination angle of 1.01° (Figure S1b)). This focal length s can be calculated geometrically, leading to $s = 34.87$ mm (Figure S2). The central stop in the condenser is incorporated into the lens model as an annular aperture whose angular range matches the range of the actual condenser opening, namely from $\theta_{min} = 0.83°$ to $\theta_{max} = 1.18°$. To achieve this angular range at a distance s from the condenser, the minimum and maximum radii of the annulus for the lens model must satisfy $R_{min\_lens} = s \tan\theta_{min} = 0.51$ mm and $R_{max\_lens} = s \tan\theta_{min} = 0.72$ mm.

This leads to the following equation for the condenser field produced by the condenser lens model [1]:

$$U_{lens-model}(r) \propto \int_{R_{\substack{min\\lens}}}^{R_{\substack{max\\lens}}} J_0\left(\frac{krR}{s}\right) R\, dR \propto \frac{R_{max\_lens}}{r} J_1\left(\frac{krR_{max\_lens}}{s}\right) - \frac{R_{min\_lens}}{r} J_1\left(\frac{krR_{min\_lens}}{s}\right) \qquad \text{(S1)}$$

with R the radial coordinate in the mean condenser plane, and r the radial coordinate for the in-focus condenser spot in the specimen plane.

The plane-wave angular spectrum of the field $U(r)$ for the annular lens model is given by its Fourier transform. In a cylindrical coordinate system, this yields

$$\hat{E}(\theta) = 2\pi \int_0^\infty U(r) J_0(rk_r)\, rdr = 2\pi \int_0^\infty U(r) J_0(rk\, sin\, \theta)\, rdr \quad \text{(S2)}$$

where $\hat{E}(\theta)$ is the angular spectrum as a function of the azimuthal illumination angle θ of the component plane wave. The θ dependence arises through the radial component k_r of the wave vector k, where $k_r = k \sin\theta$. In practice, the upper limit of infinity in the integral was replaced by a radial distance at which the field $U(r)$ is very close to zero (r_{max} =100 µm was used for the calculations in Figure S3).

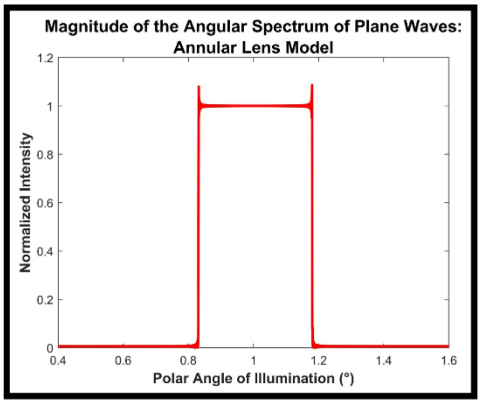

Figure S3: Equation (S2) was used to compute the angular spectrum of plane waves for the field produced by the annular lens model of the ellipsoidal mirror (Equation (S1). The resultant angular spectrum shows that the field can be described as a sum of plane waves of equal magnitude at the series of polar angles defined by the ellipsoidal mirror (0.83–1.18°). This provides a justification for using a plane-wave model for illumination of the sample. Note that we used a simplified version of this model that considers only a plane wave at the mean polar angle of illumination.

S3. Extension of the Mie theory to oblique plane waves

To account for a plane wave that forms a polar angle θ oblique to the optical axis, we developed a simple, geometrical extension of the Mie theory which is based on a non-oblique plane wave, namely a plane wave orthogonal to the *z* axis.

An oblique plane wave is always parallel to a series of oblique planes which intersect the zone plate plane, and Mie theory holds on each of these oblique planes (Figure S4a). The geometry described in the legend to Figure S4a enables calculation of the angle ψ_M between two sides R and R_M of a triangle defining points where the conventional Mie theory holds, and therefore the scattered wave $S_\theta(x', y')$ at any point (x', y') in the zone plate arising from an oblique plane wave striking the sample at the polar angle θ can be calculated:

$$R = (cos\theta)(D - y'tan\theta)\,, \qquad R_M = \sqrt{D^2 + x'^2 + y'^2}, \qquad \text{(S3)}$$
$$\psi_M = cos^{-1}(R/R_M), \qquad S_\theta(x', y') = S(\psi_M)$$

As expected, when the illuminating plane-wave is not tilted (θ=0°), the scattered field produced by the nanosphere is centered in the zone plate. When the illuminating plane wave is tilted (θ=1°), then the scattered field is shifted downward in the direction of the tilt (Figure S4b).

Thus the scattered field takes the form $U_{SW}^{zp}(x', y', z) = \frac{S_\theta(x', y')}{-ik}\left(\frac{exp(ikR_M(z))}{R_M(z)}\right)$, where R_M is a function of z through the distance $D = R_f - z$ between the nanoparticle and the zone plate, yielding Equation 11 in the main text. The plane wave field is obtained by noting that the phase of the oblique plane wave when it strikes the zone plate is given by the distance traveled R in Equation (S3) producing the field e^{ikR} corresponding to Equation 10 in the main text.

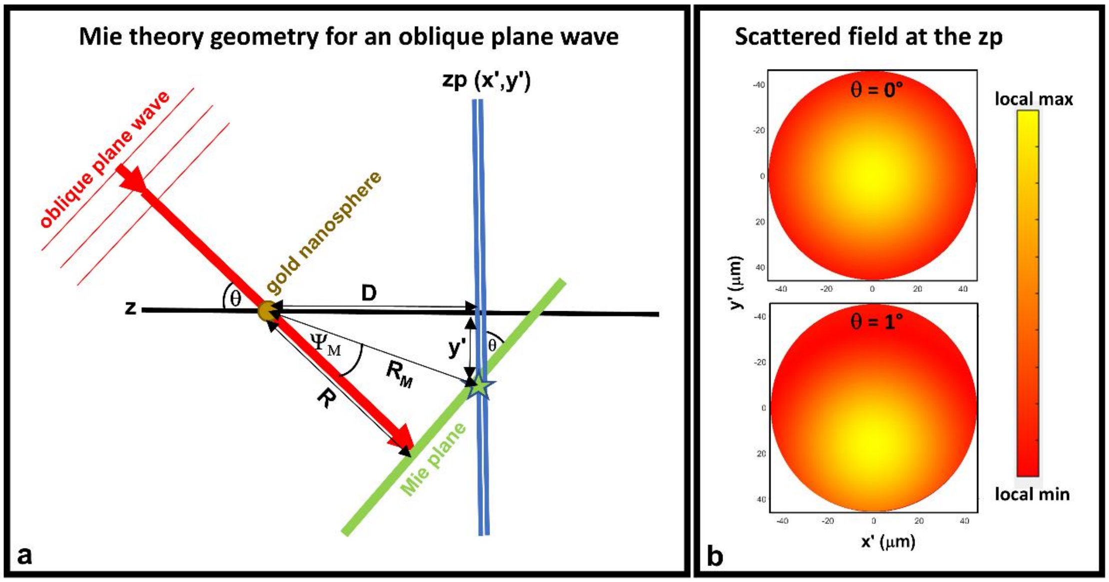

Figure S4: a.) Calculation of the Mie scattered field in the zone-plate (zp) plane due to an oblique plane wave at a polar angle θ to the optical axis (z). The gold nanosphere is situated at a distance D from the zone-plate (zp) objective plane, which is described by Cartesian coordinates (x', y'), where the x' axis is perpendicular to the page. Conventional Mie theory holds in the green Mie plane, and the scattered field there can be calculated as a function of the angle Ψ_M and the distance R_M. This Mie plane intersects the zone-plate plane along a line perpendicular to the page (the x' direction) running through the green star, and so the conventional Mie theory applies along this line. To compute the scattered wave over the full zone plate, the distance R from the nanosphere to the Mie plane is changed to generate for each value of y', a complete range of x' values for the scattered wave. b.) Shown is the magnitude of the scattered wave $S_\theta(x', y')$ as calculated by the oblique Mie theory for a 63.2 nm gold nanosphere imaged at 510 eV for both a non-tilted (θ=0°) and a tilted plane wave (θ=1°). This magnitude is calculated at a distance of 938 μm from the nanosphere corresponding to the in-focus location of the soft TXM at 510 eV, and over a circular domain corresponding to the 25 nm zone-plate dimensions (45.337 μm). Note the downward shift along y' of the image when the illumination plane wave is tilted in the y' direction (θ=1°).

S4. Simplification of the Mie theory to the anomalous diffraction approximation (ADA) for 60 nm gold nanospheres imaged with 2.4 nm light

Under certain conditions, the Mie theory for the scattered field $\boldsymbol{S}(\psi) = \left(S_1(\psi), S_2(\psi)\right)$ reduces to a scalar theory known as the anomalous diffraction approximation (ADA) [2,3]. When anomalous diffraction applies, $S_1(\psi) = S_2(\psi) = S_{ADA}(\psi)$, with the scattered field given by the much simpler expression:

$$S_{ADA}(\psi) = k^2 \int_0^a \left(1 - e^{2ik(n-1)\sqrt{a^2-\xi^2}}\right) J_0(k\xi\psi)\xi d\xi \tag{S4}$$

where ψ is the scattering angle from the nanosphere to a point on the zone plate, ξ is the radial coordinate in the nanosphere and J_0 the Bessel function of the first kind. This equation reflects the sum of the optical path length along different rays parallel to the z axis passing through the nanosphere relative to rays passing outside the nanosphere [2]. (Note that Equation (S4) has been modified from [4] by changing i to -i, since the Mie and ADA use alternate conventions for the plane wave, e^{ikz} vs e^{-ikz} respectively).

The preceding simplification for the scattered wave arises when the refractive index of the sphere is close to one, and the size of the sphere is much larger than the wavelength, conditions which should hold for soft X-ray imaging of the 60 nm nanospheres. To test this directly, we computed $S_1(\psi)$, $S_2(\psi)$ and $S_{ADA}(\psi)$ for a 60 nm – diameter gold nanosphere and verified that $S_1(\psi) = S_2(\psi) = S_{ADA}(\psi)$ (Figure S5). This demonstrates that the scalar anomalous diffraction theory applies to soft TXM imaging of the gold nanospheres examined here.

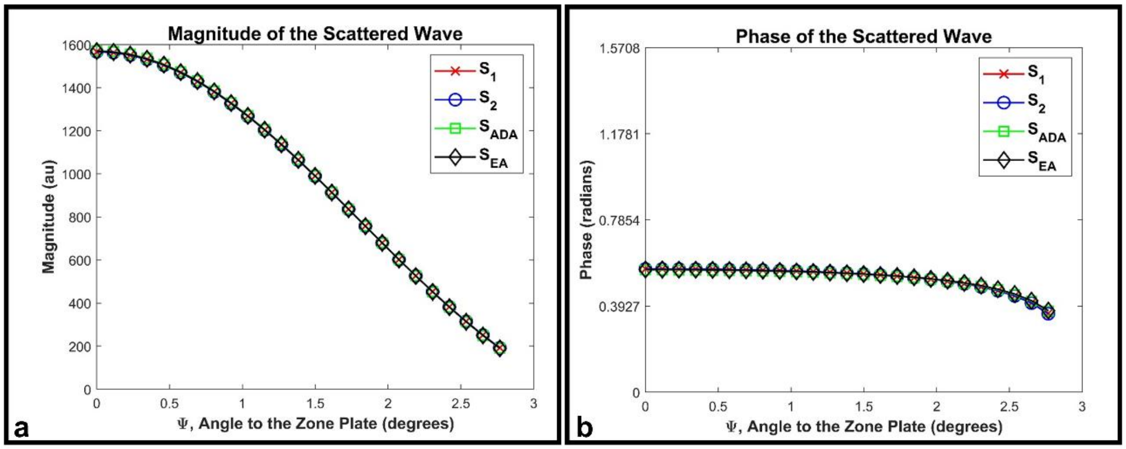

Figure S5: For 60 nm gold nanospheres imaged with 2.4 nm light, the scattered fields calculated by Mie theory ($S_1(\psi), S_2(\psi)$), by the anomalous diffraction theory ($S_{ADA}(\psi)$) and by the eikonal approximation ($S_{EA}(\psi)$) are all equivalent.

Finally, note that for all the calculations in this study, we typically computed $S_1(\psi)$ using the Mätzler Matlab code for the full Mie theory, but as demonstrated here, we could have also calculated the value for $S_2(\psi)$ or $S_{ADA}(\psi)$ since all are equivalent.

S5. Equivalency of the Mie theory and the parabolic wave equation (PWE) for 60 nm gold nanospheres imaged with 2.4 nm light

Our model uses Mie theory to propagate light through the nanosphere, and this has the advantage that the result is an exact solution to Maxwell's equations. However, for more complex samples, Selin et al. [5] have instead used the parabolic wave equation to propagate light through the sample. Here we show for the case of the 60 nm gold nanospheres that the parabolic wave equation produces a solution that is essentially identical to that of the Mie theory.

For a spherical particle, the parabolic wave equation written in cylindrical coordinates is:

$$\frac{\partial U(r,z)}{\partial z} = \frac{i}{2k}\left(\nabla^2{}_r + k^2(n^2(r,z) - 1)\right)U(r,z) \tag{S5}$$

where the complex refractive index $n = 1 - \delta + i\beta$ for $r \leq a$ and $n = 1$ for $r > a$. After rearrangement, this becomes:

$$\left(\nabla^2{}_r + 2ik\frac{\partial}{\partial z}\right)U(r,z) = W(r,z)U(r,z) \tag{S6}$$

with $W(r,z) = k^2(1 - n^2(r,z))$. This equation is the time-independent Schrödinger equation with $W(r,z)$ the reduced potential. It has been used to investigate not only the non-relativistic scattering of a particle by a potential whose size is of length a, but also light scattering by a sphere of radius a subject to the reduced potential $W(r,z) = k^2(1 - n^2(r,z))$ determined by the wavelength of light and the sphere's refractive index.

When the $\nabla^2{}_r$ term is small compared to the other two terms in Equation (S6), then it can be ignored, and the resulting partial differential equation can be easily solved. This approximation is known as the eikonal approximation, and in the case of light scattering it is valid when the wavelength is small relative to the diameter $2a$ of the sphere, and when the magnitude of the complex refractive index is close to one. Note these are the same two conditions which are also required for the Mie theory to be approximated by the anomalous diffraction approximation.

For the case of light scattering in the eikonal approximation, the solution of the first-order partial differential equation for the field is given by [6]:

$$U(r) = exp(ikz) + \frac{S(\psi)}{(-ik)}\left[\frac{exp(ikr)}{r}\right] \tag{S7}$$

with the scattering amplitude:

$$S_{EA}(\psi) = k^2 \int_0^a \left(1 - e^{ik(n^2-1)\sqrt{a^2-\xi^2}}\right) J_0(k\xi\psi)\xi d\xi \tag{S8}$$

By comparing Equation (S8) with Equation (S4), we see that the solution for the field in the eikonal approximation derived from the parabolic wave equation is nearly identical to the solution for the field in the anomalous diffraction approximation derived from the Mie theory, except the exponential term $n^2 - 1$ in the scattering amplitude $S_{EA}(\psi)$ in the eikonal approximation becomes $2(n-1)$ in $S_{ADA}(\psi)$ in the anomalous diffraction approximation. Note that these two terms are effectively equal as can be seen by expanding n^2 and dropping higher order terms in β and δ (which are each on the order of 10^{-3}). To demonstrate this directly for a 60 nm gold sphere imaged with 2.4 nm X-ray light, we have computed the solution for the scattering amplitude $S_{EA}(\psi)$ in the eikonal approximation and compared it to that for $S_{ADA}(\psi)$ in the anomalous diffraction approximation and find perfect agreement (Figure S5).

In sum, we have shown that for the imaging conditions considered here, namely a 60 nm gold nanosphere and X-ray light of 2.4 nm, the Mie theory and the parabolic wave equation yield virtually identical solutions because each can be accurately approximated by a simpler form that presumes straight line ray paths through the sphere without reflection or refraction. This result demonstrates that the propagation of light through the nanosphere is essentially identical in our model (pc-Mie) and in the Selin et al. model (pc-PWE).

S6. Calculation of the nanosphere's plane-wave and scattered-wave fields at the camera

The plane-wave field follows by direct substitution of Equation 10 into Equation 9 from the main text, and produces:

$$U_{\theta-PW}^{cam}(x,y,z) = Cexp\left(ik\left(R_f - z\right)\cos\theta\right) \times$$
$$\iint_{aperture} dx'dy' \left\{exp\left(-ik\frac{x'^2+y'^2}{2f}\right)\right\}\left\{exp\left(ik\frac{x'^2+y'^2}{2R_0}\right)exp\left(-ik\frac{xx'+(y-R_0\sin\theta)y'}{R_0}\right)\right\} \tag{S9}$$

The constant C in front of the integral arises from Fresnel diffraction:

$$C = \frac{-iexp(ikR_0)exp(ik\frac{(x^2+y^2)}{2R_0}}{\lambda R_0} = \frac{-i}{\lambda R_0}C_{oc} \tag{S10}$$

where we define C_{oc} as the oscillatory part of the constant given by the product of the two complex exponential terms.

Thus, the final equation for the plane-wave field at the camera is

$$U_{\theta-PW}^{cam}(x,y,z) = \frac{-i}{\lambda R_0}C_{oc}exp\left(ik\left(R_f - z\right)\cos\theta\right) \times$$
$$\iint_{aperture} dx'dy' \left\{exp\left(-ik\frac{x'^2+y'^2}{2f}\right)\right\} \times \tag{S11}$$
$$\left\{exp\left(ik\frac{x'^2+y'^2}{2R_0}\right)\exp\left(-ik\frac{xx'+(y-R_0\sin\theta)y'}{R_0}\right)\right\}$$

The scattered field at the camera is also calculated by direct substitution of Equation 11 into Equation 9 from the main text yielding:

$$U_{\theta-SW}^{cam}(x,y,z) = C \iint_{aperture} dx'dy' \left\{\frac{S_\theta(x',y')}{-ik}\left(\frac{exp(ikR_M)}{R_M}\right)\right\} \times \tag{S12}$$
$$\left\{exp\left(-ik\frac{x'^2+y'^2}{2f}\right)\right\} \times \left\{exp\left(ik\frac{x'^2+y'^2}{2R_0}\right)exp\left(-ik\frac{xx'+yy'}{R_0}\right)\right\}$$

However, this expression for $U^{cam}_{\theta-SW}(x,y,z)$ in Equation (S12) can be simplified with a paraxial approximation, since the maximum angle from the sample plane to the zone-plate plane is small (~3°). With $R_M = \sqrt{(R_f - z)^2 + x'^2 + y'^2}$, the term $\exp(ikR_M)/R_M$ becomes $(1/R_f) exp\, ik\left(R_f - z + \frac{x'^2+y'^2}{2R_f} + \frac{(x'^2+y'^2)z}{2R_f{}^2}\right)$, since $(R_f - z) \gg (x'^2 + y'^2)$ and since $|z| \ll R_f$, with z denoting the z shifts of the stage about the focal position R_f. With this simplification, three of the terms in the integrand in Equarion (S12) now equate to the lens law $exp\left(ik(x'^2 + y'^2)\left[\frac{1}{2R_f} + \frac{1}{2R_0} - \frac{1}{2f}\right]\right)$ and so disappear, resulting in the simplified expression presented in the main text:

$$U^{cam}_{\theta-SW}(x,y,z) = \frac{1}{2\pi R_0 R_f} exp\left(ik(R_f - z)\right) C_{oc} \times \iint_{\mathcal{A}} dx'dy' \left\{S_\theta(x',y') exp\left(ik\frac{(x'^2+y'^2)z}{2R_f{}^2}\right)\right\} exp\left(-ik\frac{xx'+yy'}{R_0}\right) \tag{S13}$$

Note that this corresponds to the Fourier transform of the bracketed pupil function, which incorporates the scattered field and wavefront aberration arising from defocus z.

Note also that the oscillatory constant term C_{oc} is present in the expressions for both the plane wave field and scattered wave field at the camera and so can be factored out. This term is also invariant to rotation about the optical axis and therefore plays no role when fields are rotated around the z axis, as occurs in the partial coherence calculations. Thus we set this term to one for all of the calculations performed in this study.

S7. Incoherent Beer's law model

Otón et al. developed the first 3D TXM model, the inc-BL model [7]. The model bears some resemblance to conventional models for fluorescence microscopy, but with fluorescence intensity replaced by the loss of intensity, namely the absorption of the light as it passes through the sample. As in models for fluorescence, the absorption at any point in the sample is convolved with the point-spread function $h(r,z)$ of the objective, and this yields the final intensity at the camera.

For the case of a nanosphere, the inc-BL model can be written in cylindrical coordinates leading to the equation in the main text:

$$I^{cam}(Mr) = 1 - \int_{-a}^{a}\left(\mu(r,z) exp\left(-\int_{-a}^{z}\mu(r,\xi)\, d\xi\right)\right)\otimes_r h(r,z) dz \tag{S14}$$

with M the microscope magnification and μ the linear absorption coefficient defined for the case of a nanosphere with radius a by:

$$\mu(r,\xi) = \begin{cases} 2k\beta & r^2 + \xi^2 \le a^2 \\ 0 & r^2 + \xi^2 > a^2 \end{cases} \tag{S15}$$

The point-spread function is obtained by first computing the field arising from a lens whose NA equals that of the zone plate:

$$U^{cam}_{lens}(\rho_{obj}, z) \propto \int_0^{r_{zp}} rdr\, exp\left(ik\frac{r^2 z}{2R_f{}^2}\right) J_0\left(\frac{k\rho_{obj} r}{R_f}\right) \tag{S16}$$

Here the radius of the lens equals that of the zone plate r_{zp}, z is the amount of defocus and ρ_{obj} is the radial coordinate of the demagnified image (the coordinate for the image in "object-space" units). Note that this equation is a polar coordinate version of the integral equation for the scattered wave (Equation 13) in the main text, but with the scattered wave amplitude S set to one. This reflects the fact that both the inc-BL model and the pc-Mie model treat the zone plate as a lens with defocus.

The point-spread function $h(r,z)$ is then computed from the squared magnitude of the field $U^{cam}_{lens}(\rho_{obj}, z)$ in Equation (S16). Then this intensity point-spread function is used to compute the intensity $I^{cam}(Mr)$ from Equation (S14) when the nanosphere is in focus. To obtain the full image of the nanosphere in z, the point spread function is shifted in z by the amount of defocus, and Equation (S14) is reapplied.

For the case of an apodized objective used to simulate an annular version of the inc-BL model in Figure 3 in the main text, we substituted $0.7 \times r_{zp}$ for the lower limit of integration in Equation (S16). The factor 0.7 corresponds to the ratio of the minimum to maximum polar angles of illumination (0.83/1.18), and so provides a proxy for the fraction of annular illumination in the pc-Mie model.

S8. Effect of coherence patch size in the mean-angle condenser model

The results for the pc-Mie model presented in Figure 2 of the main text were obtained with a mean polar angle of the condenser illumination ($\theta_m = 1.01°$) and a small azimuthal-angle coherence patch size ($p_\varphi = 1°$). We also computed the predicted images of the pc-Mie model for larger coherence patches by adding fields within azimuthal angular sectors of 6°, 12° and 36° before computing the intensity of each azimuthal sector and then adding it to the intensity of 360 overlapping sectors that spanned the full circumference of the ellipsoidal mirror (Figure S6).

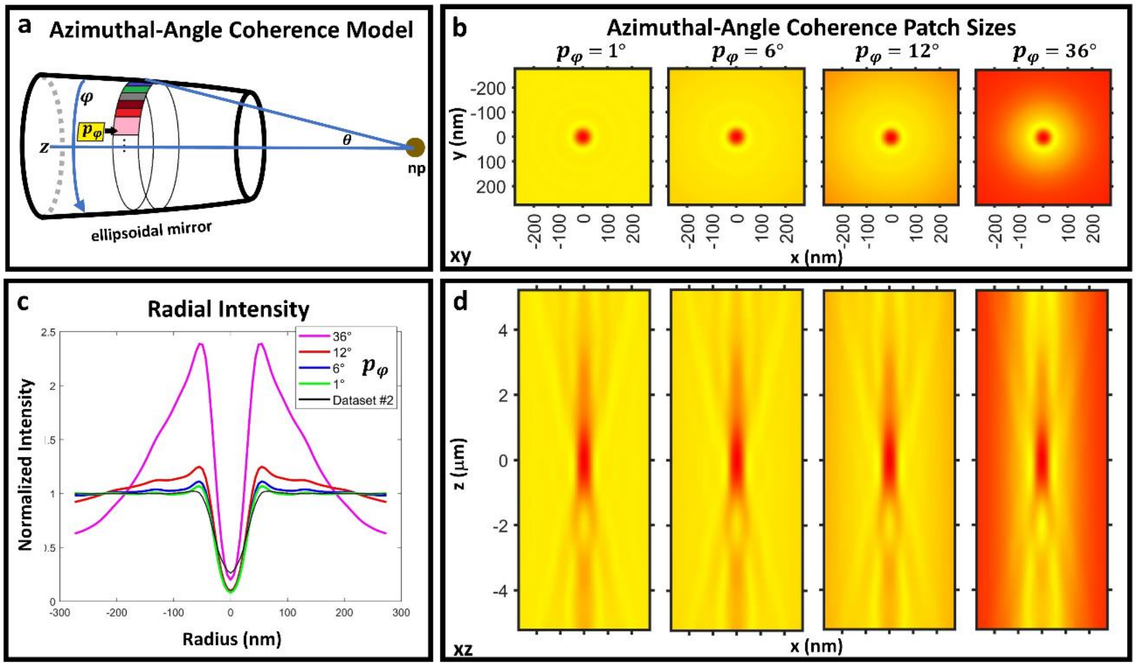

Figure S6: Effects of azimuthal coherence patch size in a mean-angle condenser, where the full ellipsoidal mirror is replaced by a ring of coherence patches at the mean angle of illumination (1.01°). The schematic (a) illustrates the incremental shifting of an azimuthal angle coherence patch ($p_\varphi \sim 10°$ in the schematic) around the mirror circular cross section. As the azimuthal angular patch size p_φ increases in the pc-Mie model, the predicted images (b,d) show marked changes in contrast levels. These arise from increased ringing around the nanosphere perimeter with increased patch sizes (c).

We found that as the azimuthal coherence patch size increased, the contrast range for the predicted images shifted significantly (Figure S6b,d). To better understand these changes, we computed radial intensity profiles through the center of the nanosphere images. The nanosphere center was defined as the minumum-intensity pixel in the 3D dataset, and the focal plane containing this minimum-intensity pixel was defined as best focus. We then computed an average radial intensity in this best-focus plane for the experimental data and compared it to the model predictions with different coherence patch sizes.

These radial intensity profiles (Figure 6c) revealed that larger azimuthal-angle coherence patch sizes led to increasingly larger overshoots around the edge of the nanosphere. These overshoots increased the total intensity range of the image thereby producing the observed altered contrast ranges for larger coherence patch sizes (Figure 6b,d). By comparison, we found that patch sizes below $p_\varphi = 1°$ led to virtually identical radial intensity profiles. Furthermore, these profiles from smaller coherence patches provided the best match to the measured data. This result suggests that the effective azimuthal-angle coherence patch size on the microscope is $p_\varphi \leq 1°$.

S9. Rotation of the experimental data around the optical axis

We found we could eliminate the day-to-day differences in axial asymmetry observed in Figure 2 in the main text by rotating each experimental dataset around the optical axis by a different angle. We estimated this angle by examining slightly defocused xy images, each of which exhibited a localized bright spot, but each at a different angle in the first defocussed diffraction ring (Figure S7a). By rotating each dataset to position this bright spot at 6 PM (Figure S7b), we obtained very similar axial profiles for the three experimental datasets (Figure 4a in the main text).

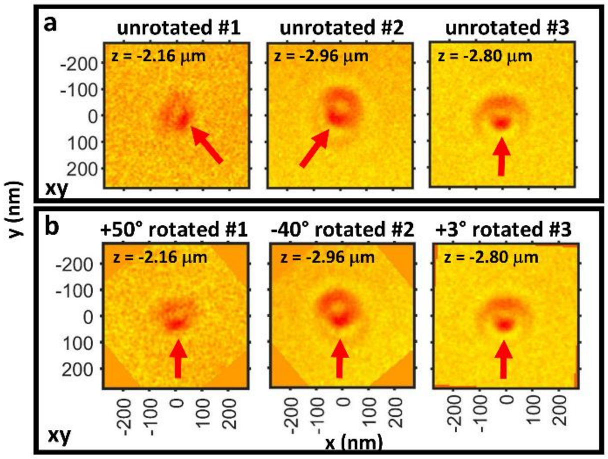

Figure S7: Asymmetry in the diffraction ring pattern. a.) Red arrows point to highly absorbing spots in the first defocussed diffraction rings of the three datasets. b.) The different datasets were rotated by the amounts indicated to position the absorbing spot at the same angular location (red arrows, here ~6 PM, rotated #1-#3). This rotation then eliminated most of the day-to-day variation observed in the experimental data.

S10. Estimation of the axial tilt in the data

We estimated the axial tilt of the experimental data by manually drawing a line along the apparent tilt axis of the xz and yz views of the data, after it had been rotated around the optical axis such that the asymmetry present in the first defocussed diffraction ring was positioned at 6 PM (Figure S7). See the legend for Figure S8 for details of the axial tilt estimation procedure.

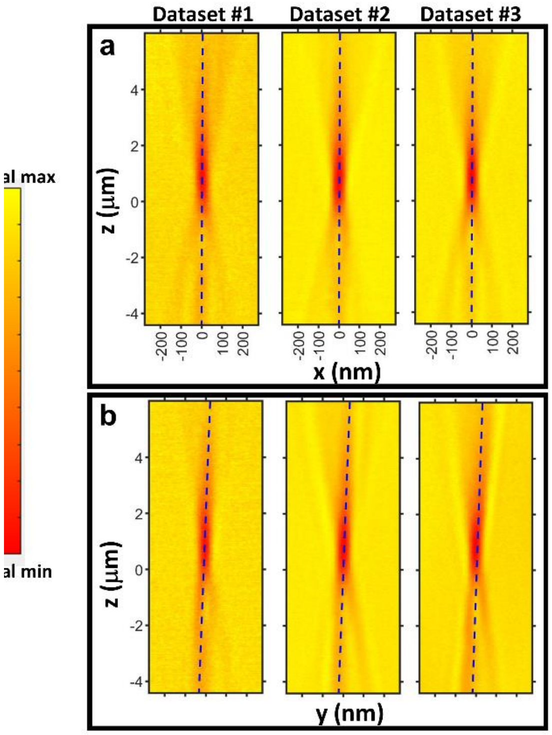

Figure S8: Estimation of axial tilt angles from data acquired on three different days. Each 3D image of a 60 nm gold nanosphere was first rotated about the *z* axis to align the asymmetry in the out-of-focus diffraction rings (Dataset #1: 50°, Dataset #2: -40°, Dataset #3: 3° - see Figure S7). Next, axial xz and yz slices of the rotated nanosphere images were generated (each containing the minimum-intensity pixel in the 3D volume). Then, the axial tilt along xz and yz was estimated by eye by following along *z* the defocussed center of the nanosphere as defined by the largest absorption (red intensity). The vertical line (blue dashes) shows this estimated tilt for each dataset in both *xz* and *yz*. The blue-dashed lines were significantly more tilted in the *yz* slices (bottom) compared to the *xz* slices (top). To calculate average axial tilt angles, we computed the arctangent of the slope of the blue dashed lines ($tan^{-1}(\Delta x/\Delta z)$ and $tan^{-1}(\Delta y/\Delta z)$). This yielded an average tilt of 0.026° in *xz* and 0.26° in *yz*, showing that tilt was approximately an order of magnitude larger along *y* compared to *x*.

S11. Calculation of polar tilt angle as a function of azimuthal angle in a tilted condenser

We use the simplified mean-angle condenser configuration in which azimuthal illumination occurs at a single angle, namely $\theta_{mean} = 1.01°$ (Figure S1b). In Figure S2, we calculated the radius (y_0) and distance to the sample (s) of this simplified mean-angle condenser. We now consider the effect of tilting the incoming beam and this simplified condenser with respect to the optical axis defined by the zone plate and camera by the tilt angle ω_{tilt} ($\omega_{tilt} \approx 0.26°$, as estimated from the measured data (Figure S8)). For such a tilted condenser, the polar angle θ for illumination of the sample changes as a function of the azimuthal angle φ around the condenser perimeter. To compute $\theta = \theta(\varphi)$, the geometry of the tilted condenser must be considered (Figure S9). The resultant formula for $\theta = \theta(\varphi)$ is derived in the legend to Figure S9.

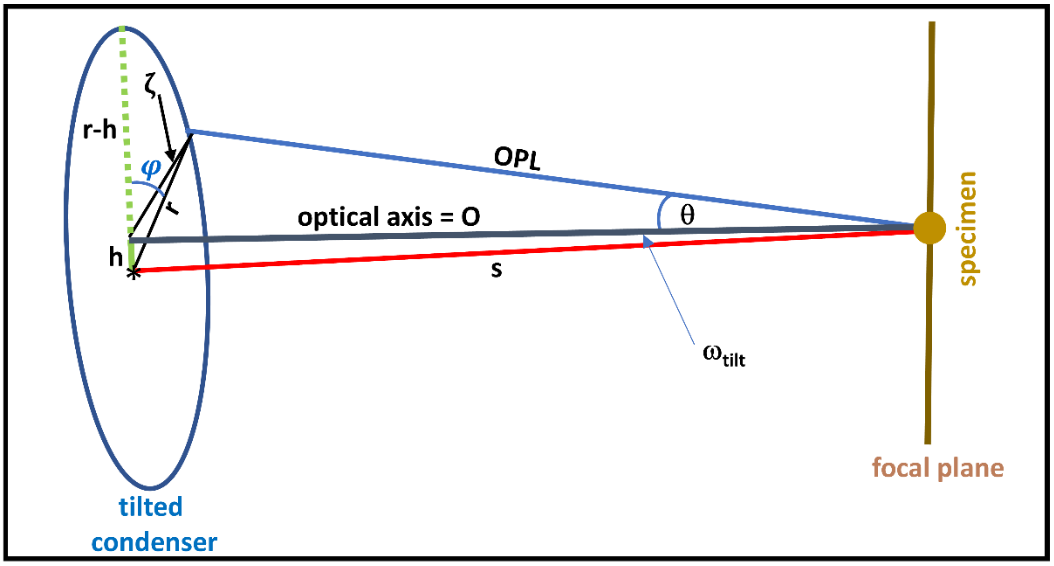

Figure S9: Shown is a condenser tilted at an angle ω_{tilt} relative to the optical axis defined by the zone plate and camera ($\omega_{tilt} \approx 0.26°$ as estimated from the measured images (Figure S8)). The condenser plane is perpendicular to the line s whose length is the focal length of the equivalent lens. We must calculate the polar angle of illumination θ as a function of the azimuthal angle φ around the condenser perimeter. This can be accomplished by a double application of the law of cosines. We first consider the triangle with sides ζ, h, r. We know h from the right triangle determined by the tilt: $h = s * tan(\omega_{tilt})$, where $s = 34.87$ mm (Figure S2). We know $r = 0.62$ mm (the radius r is given by y_0 in Figure S2)). From this, we apply the law of cosines the first time to obtain ζ as a function of φ: $\zeta(\varphi) = \sqrt{h^2 + r^2 - 2hr\cos\varphi}$. Next, we consider the second triangle $\zeta(\varphi), O, OPL$. O is given simply from the right triangle, $O = s/\cos(\omega_{tilt})$, and OPL is also defined by another right triangle : $OPL = \sqrt{s^2 + r^2}$. Thus we know all sides of this second triangle, and applying the law of cosines a second time gives $\zeta(\varphi)^2 = O^2 + OPL^2 - 2O * OPL * \cos\theta$, which yields the desired relationship $\theta = \theta(\varphi)$, and the plot shown in Figure 4d in the main text.

S12. Radial intensity profiles of the tilted vs non-tilted theory

Compared to the non-tilted theory, the tilted condenser theory exhibitted slightly different radial intensity profiles along the direction of tilt (y - Figure S10a) but not in the non-tilted direction (x – Figure S10b). However, this small difference along the y axis was averaged out when computing the radial intensity from the tilted theory (Figure S10c), so the computationally simpler non-tilted theory was used for all quantitative comparisons.

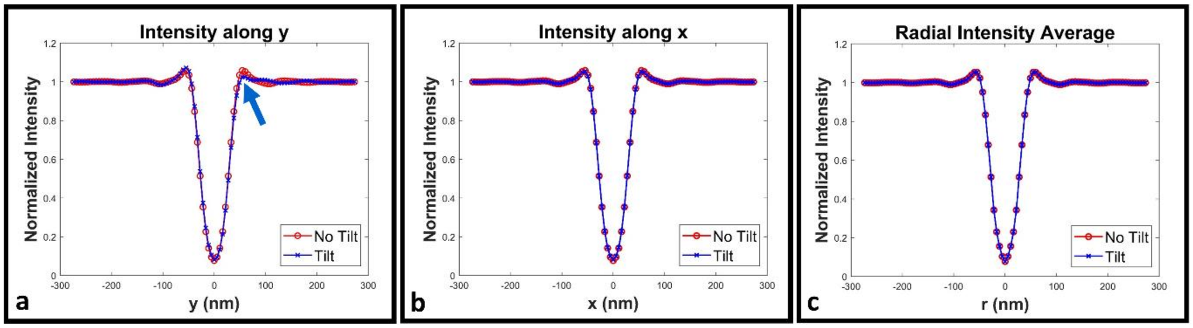

Figure S10: Comparison of in-focus intensity profiles between the non-tilted (red) and tilted (blue) versions of the mean-angle, plane-wave condenser model. The tilt is introduced in the *y* direction, and this yields a small asymmetry in the intensity profile along *y* as compared to the untilted model (a, blue arrow). The difference is small in the *y* direction because the tilt itself is small, and this small tilt only becomes obvious in the image as one moves far out-of-focus. In the *x* direction, there is no difference in the intensity profiles between the tilted and non-tilted models (b), as expected because the tilt is orthogonal to *x*.There is also no difference in the radial intensity profiles between the tilted and non-tilted condensers (c). This is because the small difference arising in the *y* direction is averaged out when computing the mean radial intensity around the nanosphere center point.

S13. Structural characterization of the 60 nm gold nanospheres by SEM and TEM

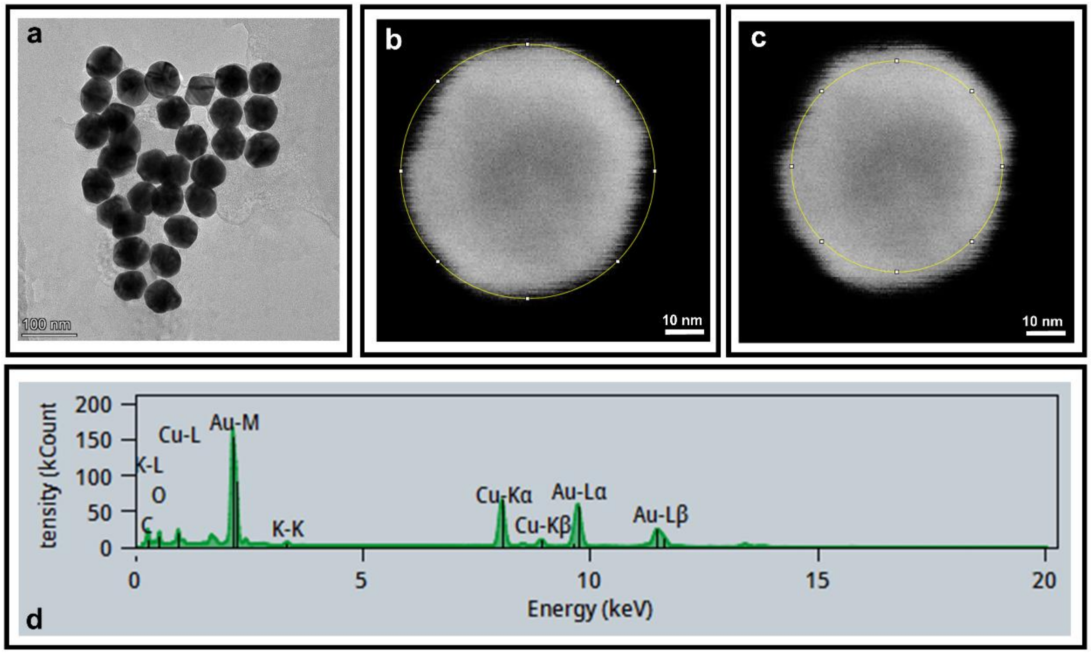

Figure S11: (a) TEM overview images of the 60 nm gold nanoparticles showed that they were reasonably homogeneous in shape and size, with some outliers. The density of the nanoparticles is also relatively homogeneous, with some striping indicative of channeling arising from an underlying crystalline structure. We imaged the same gold nanoparticles by SEM, and measured the diameter of 35 different nanospheres by drawing a circle around each nanosphere to determine its circumference. Note that nanoparticles that were premeasured by SEM for soft TXM imaging (such as the one shown in (b,c)) were selected to be roughly spherical and of the expected ~60 nm size. To estimate the error in the diameter measurement, we also drew two extreme circles around the nanosphere (shown in (b,c): one that circumscribed (b) and one that inscribed (c) the perimeter of the nanoparticle (shown is the 63.2 nm diameter nanosphere, Dataset #2 in the main text). For this nanoparticle, the two extreme circles led to an upper and lower bound for the nanoparticle diameter of 58.0 – 67.5 nm. (d) STEM-EDX analysis revealed that the predominant element in the nanoparticle was gold, with very small contamination of oxygen, carbon and potassium. These trace amounts contribute little to the absorption of the nanosphere. The presence of copper is due to the copper grids used for imaging.

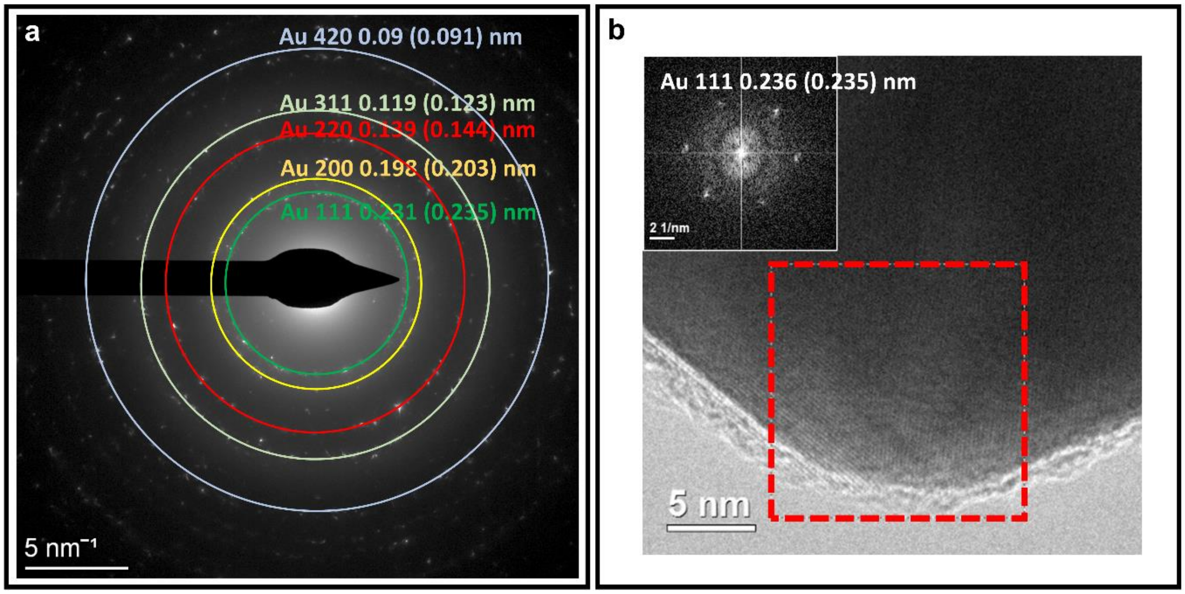

Figure S12: (a) Electron diffraction images of the gold nanospheres demonstrated that the nanospheres contain domains with crystalline gold. The white spots correspond to the expected lattice patterns for crystalline gold. Different lattice planes are shown with the corresponding measured and reference values (ICSD 163723) for the lattice spacing in parentheses. (b) High resolution TEM

images show that order domains dominate the nanoparticles. The Fourier transform from a sub-region of the area marked in red reveals the presence of a lattice that again corresponds to the one expected from crystaline gold. From the combination of these two types of measurments, we deduce that the nanoparticles are predominantly crystalline and that the crystalline domains have the density of crystalline gold.

S14. Sensitivity of radial intensity profiles to deviations in nanosphere geometry

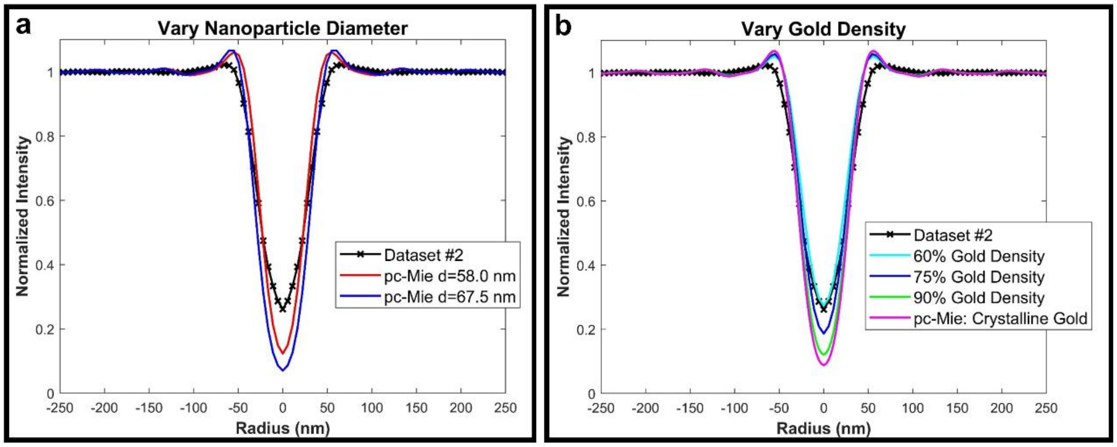

Figure S13: Neither variations in nanosphere diameter or density can explain the deviations from the measured data. Shown in both plots is the measured radial intensity profile for Dataset #2, the 63.2 nm diameter nanosphere. (a) The limiting diameters measured for this nanosphere, 58.0 nm and 67.5 nm (see the circumscribed and inscribed circles in Figure S11b,c), yield radial intensity profiles that still differ significantly from the soft TXM measurement. (b) Similarly, the measured data could only be matched if the gold density in the nanoparticle was 60% of that expected for crystalline gold, although the TEM images indicate that the gold nanosphere is in fact crystalline (see Figure S12).

References

(1) Born, M.; Wolf, E. *Principles of Optics: Electromagnetic Theory of Propagation, Interference and Diffraction of Light (7th Expanded Edition)*; Cambridge University: Cambridge ; New York, 1999

(2) Hulst, H. C. van de. *Light Scattering by Small Particles*; Dover Publications: New York, 1981

(3) Thomas, M. E. *Proc SPIE 10750 Reflect. Scatt. Diffr. Surf. VI* **2018**, *10750*. doi:10.1117/12.2323110

(4) Kokhanovsky, A. A. *Optics of Light Scattering Media: Problems and Solutions*; Wiley-Praxis series in atmospheric physics and climatology; John Wiley : Praxis: Chichester ; New York, 1999

(5) Selin, M.; Fogelqvist, E.; Holmberg, A.; Guttmann, P.; Vogt, U.; Hertz, H. M. *Opt. Express* **2014**, *22*, 30756. doi:10.1364/OE.22.030756

(6) Sharma, S. K.; Somerford, D. J. Scattering of Light in the Eikonal Approximation. In *Prog. in Optics XXXIX*; Elsevier, 1999; pp 213–290

(7) Otón, J.; Sorzano, C. O. S.; Pereiro, E.; Cuenca-Alba, J.; Navarro, R.; Carazo, J. M.; Marabini, R. *J. Struct. Biol.* **2012**, *178*, 29–37
